# In Silico Elucidation of the Molecular Mechanism Defining the Adverse Effect of Selective Estrogen Receptor Modulators

**DOI:** 10.1371/journal.pcbi.0030217

**Published:** 2007-11-30

**Authors:** Lei Xie, Jian Wang, Philip E Bourne

**Affiliations:** 1 San Diego Supercomputer Center, University of California San Diego, La Jolla, California, United States of America; 2 Bioinformatics Program, University of California San Diego, La Jolla, California, United States of America; 3 Skaggs School of Pharmacy and Pharmaceutical Sciences, University of California San Diego, La Jolla, California, United States of America; University of California San Diego, United States of America

## Abstract

Early identification of adverse effect of preclinical and commercial drugs is crucial in developing highly efficient therapeutics, since unexpected adverse drug effects account for one-third of all drug failures in drug development. To correlate protein–drug interactions at the molecule level with their clinical outcomes at the organism level, we have developed an integrated approach to studying protein–ligand interactions on a structural proteome-wide scale by combining protein functional site similarity search, small molecule screening, and protein–ligand binding affinity profile analysis. By applying this methodology, we have elucidated a possible molecular mechanism for the previously observed, but molecularly uncharacterized, side effect of selective estrogen receptor modulators (SERMs). The side effect involves the inhibition of the Sacroplasmic Reticulum Ca2+ ion channel ATPase protein (SERCA) transmembrane domain. The prediction provides molecular insight into reducing the adverse effect of SERMs and is supported by clinical and in vitro observations. The strategy used in this case study is being applied to discover off-targets for other commercially available pharmaceuticals. The process can be included in a drug discovery pipeline in an effort to optimize drug leads and reduce unwanted side effects.

## Author Summary

Early identification of the side effects of preclinical and commercial drugs is crucial in developing highly efficient therapeutics, as unexpected side effects account for one-third of all drug failures in drug development and lead to drugs being withdrawn from the market. Compared with the experimental identification of off-target proteins that cause side effects, computational approaches not only save time and costs by providing a candidate list of potential off-targets, but also provide insight into understanding the molecular mechanisms of protein–drug interactions. In this paper we describe an integrated approach to identifying similar drug binding pockets across protein families that have different global shapes. In a case study, we elucidate a possible molecular mechanism for the observed side effects of selective estrogen receptor modulators (SERMs), which are widely used to treat and prevent breast cancer and other diseases. The prediction provides molecular insight into reducing the side effects of SERMs and is supported by clinical and biochemical observations. The strategy used in this case study is being applied to discover off-targets for other commercially available pharmaceuticals and to repurpose existing safe pharmaceuticals to treat different diseases. The process can be included in a drug discovery pipeline in an effort to optimize drug leads, reduce unwanted side effects, and accelerate development of new drugs.

## Introduction

Early identification of the adverse effects of preclinical and commercial drugs is crucial in developing highly efficient therapeutics, since unexpected adverse drug effects contribute to one-third of all drug failures in the late stage of drug development [1]. Conventional practices for identifying off-targets rely on a counterscreen of compounds against a large number of enzymes and receptors in vitro [2–4]. Computational approaches could not only save time and costs spent during in vitro screening by providing a candidate list of potential off-targets but also provide insight into understanding the molecular mechanisms of protein–drug interactions. It has been shown that potential off-targets can be identified in silico by establishing the structure–activity relationship of small molecules [5–12]. However, the success of ligand-based methods strongly depends on the availability and coverage of the chemical structures used in training, and few of them directly take the target 3D structure into account. Although the assessment of protein–ligand interactions by docking studies at the atomic level is extremely valuable for understanding the molecular mechanism of adverse therapeutic effects [13,14], protein–ligand docking on a large scale is hindered by the biased structural coverage of the human proteome [15] and a lack of practical methodologies to accurately estimate the binding affinity [16]. Here we approach the problem from a different direction by postulating that proteins with similar binding sites are likely to bind to similar ligands [17]. In this study we test this postulate by predicting potential off-target binding sites for selective estrogen receptor modulators (SERMs). Several commercial drugs targeting estrogen receptor alpha (ERα) have been developed to treat breast cancers and other diseases [18]. However, therapy from these drugs such as Tamoxifen (IUPAC name: (*Z*)-2-[4-(1,2-diphenylbut-1-enyl)phenoxy]-*N*,*N*-dimethyl-ethanamine) (TAM) is associated with undesirable side effects such as cardiac abnormalities [19], thromboembolic disorders [20], and ocular toxicity [21]. To identify off-targets of these SERMs and to attempt to elucidate the molecular mechanisms explaining their adverse effects, we searched for similar ligand binding sites across fold and functional space using a template for the known SERM binding site in ERα (Protein Data Bank id: 1XPC). The search used a robust and scalable functional site prediction and comparison algorithm developed recently in our laboratory [22; Xie and Bourne, submitted]. Consequently, a similar inhibitor site is detected for Sacroplasmic Reticulum (SR) Ca2+ ion channel ATPase protein (SERCA). The prediction is further verified with detailed protein–ligand docking and surface electrostatic potential analysis. Our prediction correlates well with clinical and biochemical observations, providing molecular insight into reducing the adverse effect of SERMs. The strategy used in this case study could be applied to discover off-targets for other commercially available pharmaceuticals and to repurpose existing drugs to treat different diseases [Xie, Kinnings, and Bourne, in preparation]. The process could also be included in a drug discovery and development pipeline in an effort to optimize drug leads and reduce unwanted side effects.

## Results

We first selected 10,730 structures from the RCSB Protein Data Bank (PDB) [23] as available structural models from the human proteome by mapping sequences of all PDB structures to Ensembl human proteins using a sequence identity above 95% (see Methods). This resulted in non-human proteins being included, but with this high level of sequence identity, similarity of structure, and binding site in particular, can be assumed. These structures form 2,586 sequence clusters using a sequence identity of 30%. Of the 10,730 structures, we determined that 1,585 belong to the existing druggable proteome and correspond to 929 unique drug targets (see Methods). Using a sequence identity cutoff of 30%, 825 structures are chosen as the representative set of human druggable proteins. It is estimated that the 825 structures represent approximately 40% of the known existing druggable targets and 10% of the human druggable genome (Table S1). However, we estimate that, based on sequence similarity, and hence structure similarity, six human proteins can be mapped to one drug target. Thus, by taking homology models into account, we estimate that the structural coverage of the druggable human genome is approximately 40% (see Methods). Thus, while no means complete, we have a significant number of secondary protein targets that can be analyzed for off-site binding. In our case study, this was sufficient to find a candidate secondary target.

These 825 structures were scanned for similarity to the ligand binding site of ERα (PDB id: 1XPC) with our sequence order independent profile–profile alignment algorithm (SOIPPA) [Xie and Bourne, submitted]. A Sarcoplasmic Reticulum (SR) Ca2+ ion channel ATPase protein (SERCA) (PDB id: 1WPE) showed the most significant similarity with *p*-value < 0.001. The significance of SERCA was confirmed using a search with a larger set of 2,586 non-redundant human homologous proteins that included both druggable and non-druggable proteins (Figure S1). Besides SERCA, several other proteins exhibited significant similarity to the ERα ligand binding sites (Table S2). These sites are the subject of an ongoing investigation. It is noted that the SERCA structure 1WPE is from Oryctolagus cuniculus (rabbit), as the human SERCA is absent from the PDB. A BLAST [24] search against the Ensembl version of the human genome [25] revealed that human and rabbit SERCA share 96% sequence identity without insertion or deletion. Moreover, the transmembrane domains and known ligand binding site residues were found to share 98% and 100% sequence identity, respectively (Figure S2). Therefore, the rabbit SERCA structure was used as a reasonable structural model for human SERCA throughout this study.

SERCA plays a key role in regulating cytosolic calcium levels by accumulating calcium in the lumen [26]. SERCA consists of four SCOP domains [27]: a double-stranded beta-helix; a HAD-like domain; an ATP-binding domain N of metal cation-transporting ATPase; and a transmembrane domain M with an up–down bundle architecture. The predicated binding site is located in the all-helix transmembrane domain. It is noted that the ERα ligand binding domain itself adopts a similar all-helical orthogonal bundle architecture, but its similarity to SERCA cannot be established directly from structural comparison since the rmsd is 5.8 Å, the Z-score 3.7, and the sequence identity 7.1% as determined by CE alignment [28].

**Figure 1:**
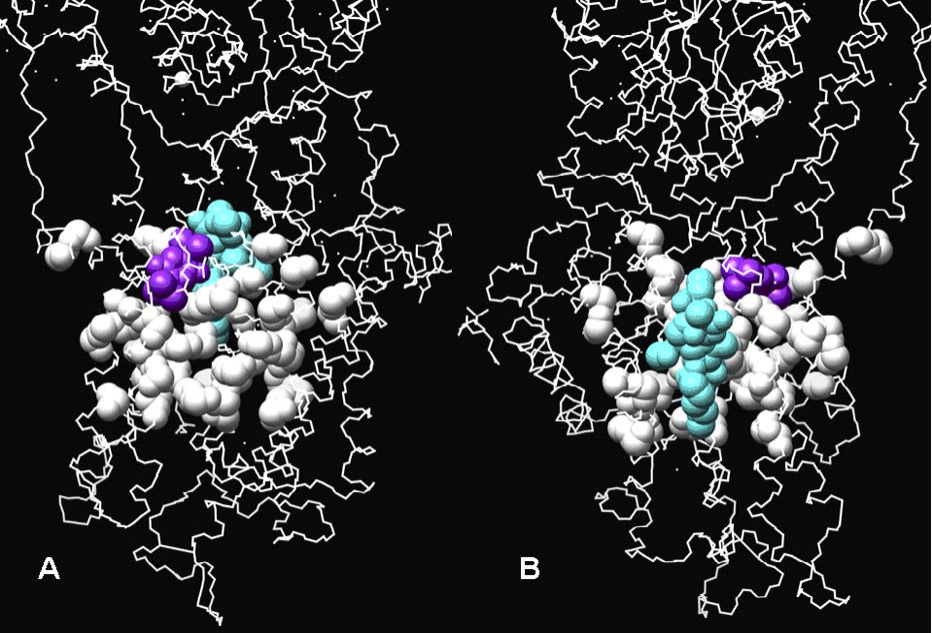
Comparison of the Predicted SERCA Ligand Binding Site with That of Known SERCA Inhibitors TG1 and BHQ The predicted binding site is represented by white spheres, BHQ by purple spheres, and TG1 by cyan spheres. (A) and (B) are two different perspectives centered on BHQ and TG1, respectively.

**Figure 2:**
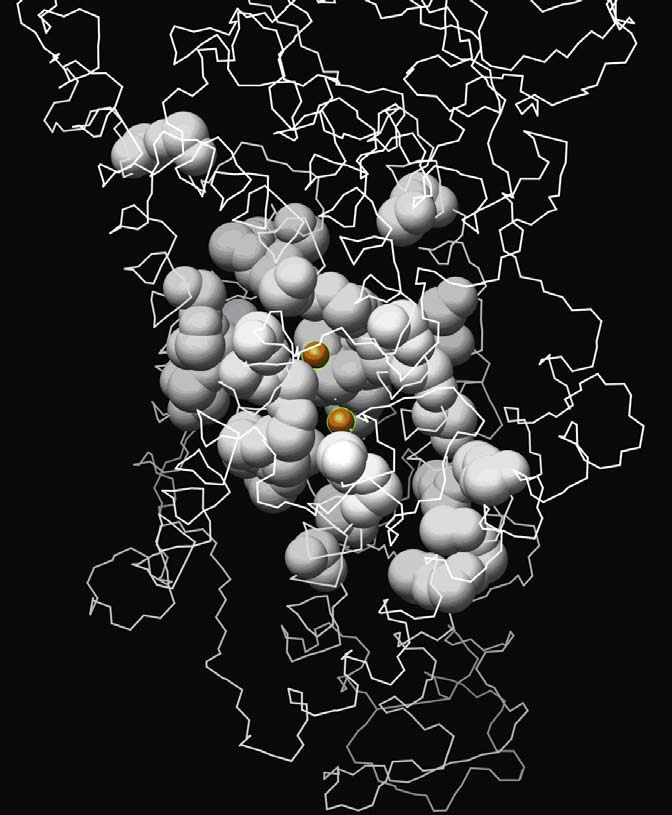
Comparison of the Predicted SERCA Ligand Binding Site (White Spheres) and the Two Calcium Ions (Orange Spheres)

A search through protein–ligand complex structures in the PDB reveals that two co-crystal inhibitors, thapsigargin (TG1) and 2,5-ditert-butylbenzene-1,4-diol (BHQ) (PDB id: 2AGV) [29] bind in the vicinity of the predicted binding sites and are in contact with part of the predicted site (Figure 1). If amino acid residues whose atomic distances to the inhibitors are less than 6.0 Å are considered as the binding site, 30% of residues overlap between known and predicated site. Thus SERM is predicted to bind to a site similar to these two inhibitors. It is suggested that the two calcium ions, which bind in the region of the putative binding site (Figure 2), are prevented from binding by SERM with consequences that are outlined subsequently.

**Figure 3:**
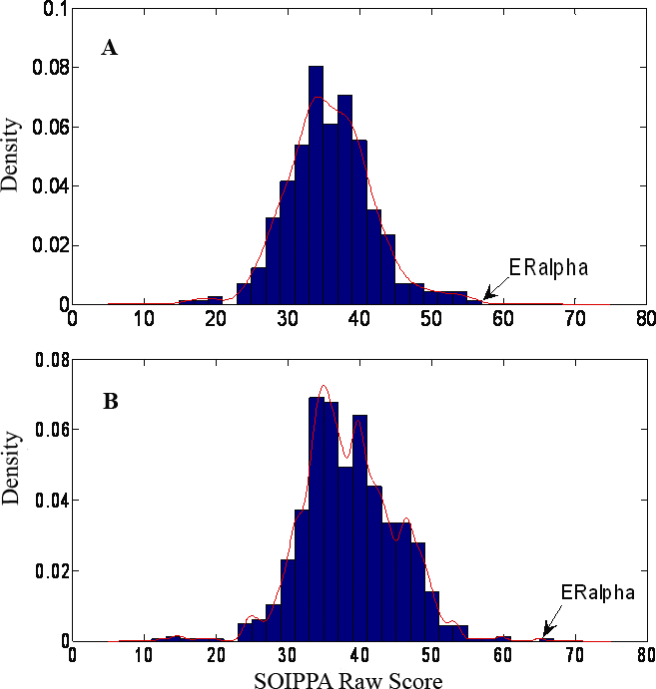
Distribution of Binding Site Similarity Scores from Searching 825 Representative Structures against SERCA for BHQ (A) and TG1 (B) Sites, Respectively The ERα is ranked top in both cases as shown by the arrows.

**Figure 4:**
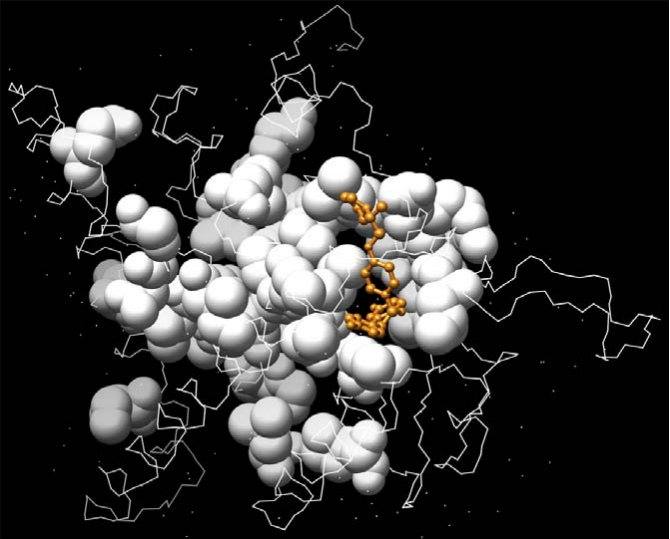
Predicated ERα Ligand Binding Site from Reverse Search by Querying the SERCA TG1 Site (White Spheres) The known bound ligand is shown in a ball-and-stick representation (gold).

In a reverse search, we scanned the set of proteins comprising the druggable proteome against the TG1 and BHQ sites of SERCA. ERα receptors were ranked at the top with a *p*-value < 0.001 for the TG1 site but a *p*-value of 0.052 for the BHQ site (Figure 3). Figure 4 illustrates that the SERM binding site is part of the predicated site. This complementarity of binding confirms the similarity between the SERCA inhibitor and the SERM binding site with high confidence.

**Figure 5:**
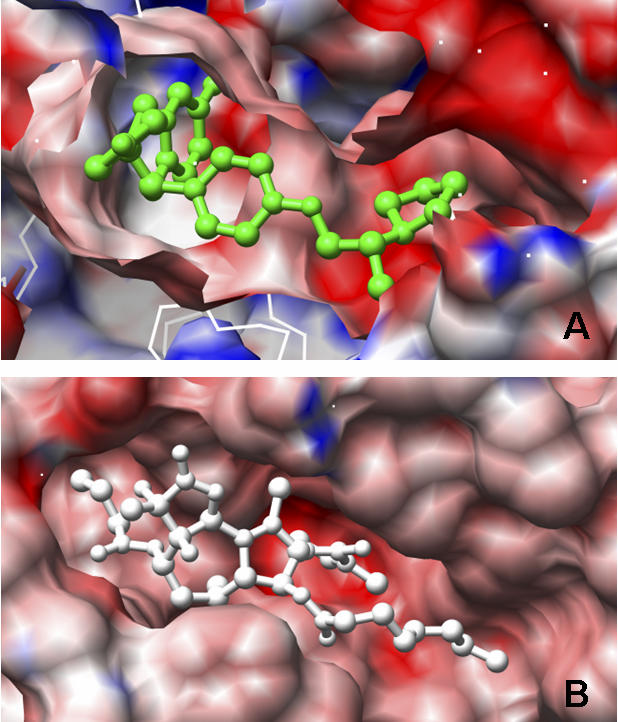
Electrostatic Potential (ES) of the Ligand Binding Site in: (A) the Original Drug Target ERα (PDB id: 1XPC), and (B) Predicated Off-Target SERCA (PDB id: 2AGV) The surface is colored according to the electrostatic potential calculated from APBS [60]. Part of the surface that covers the binding site in 1XPC is removed for better visualization. The green stick model is the co-crystallized ligand (2S,3R)-3-(4-hydroxyphenyl)-2-(4-{[(2R)-2-pyrrolidin-1-ylpropyl]oxy}phenyl)-2,3-dihydro-1,4-benzoxathiin-6-ol (AIT). The white stick model is the co-crystallized ligand thapsigargin (TG1). The color scale is set from −30 to 30 kT/e using a linear scale to elucidate ES around the ligand binding sites.

As a further test, we compared the electrostatic potential (ES) between the binding site in ERα and SERCA, as ES is an important identifier of ligand binding [30]. As seen in Figure 5, the SERM binding site is relatively negatively charged. Similarly, the binding pocket of SERCA also shows a negative potential. These observations are consistent with the binding site similarity predicated from SOIPPA at the residue level.

**Figure 6:**
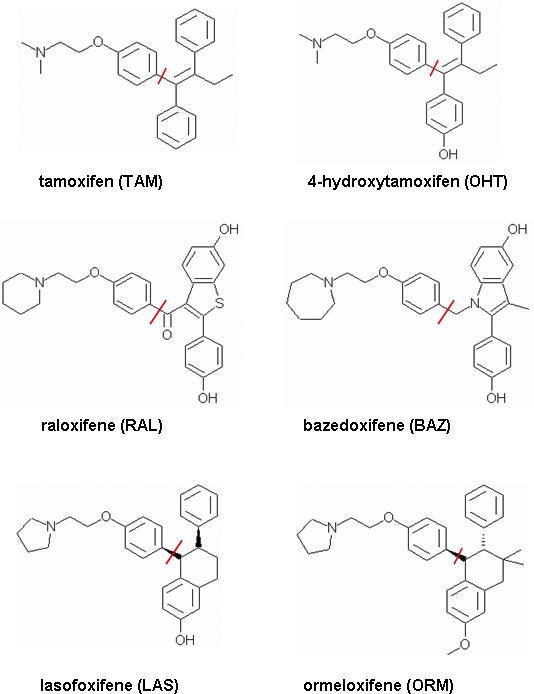
Six Selective Estrogen Receptor Modulators Either Commercially Available or in Clinical Trial N- and C-moieties are broken down by bonds marked with red bars and on the left and the right sides of 2D schema, respectively.

**Figure 7:**
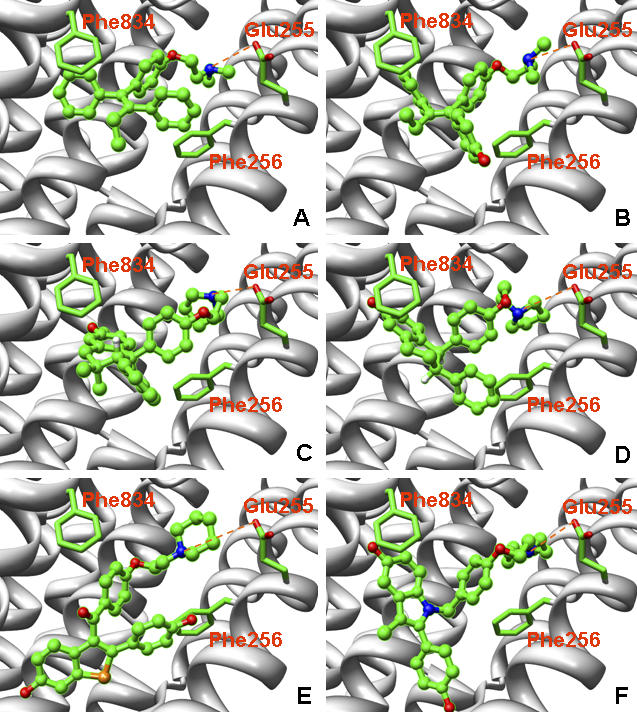
Docking Poses of Six SERMs at the SERCA TG1 Site (A) TAM, (B) OHT, (C) ORM, (D) LAS, (E) RAL, and (F) BAZ. SERCA is represented as a white backbone. Side chains of Phe256/834 and Glu255 are represented with stick models. SERMs are represented as ball and stick models. Carbon atoms are colored green; oxygens red; nitrogens blue; sulphur orange. The potential salt bridge interaction between the amine and Glu255 is indicated by an orange dashed line.

To understand the molecular mechanism of SERM's inhibition on SERCA, we performed a detailed protein–ligand interaction study by docking a series of SERM molecules to both SERCA and ERα proteins with eHits 6.2 [31] and Surflex 2.1 [32] docking software. These two free software packages were selected because of their relatively high accuracy, speed, and ease of use in a large-scale study [33,34]. Moreover, these two packages adopt different strategies during conformational search, offering independent confirmation of our findings. The conformational search in eHits is performed by breaking molecules into rigid fragments and docking them independently. The final binding pose is determined by linkage and optimization of the reconstructed ligands from the fragments. The conformational search in Surflex relies on generation of an idealized binding site ligand called protomol and alignment of the ligand to the protomol to achieve maximum molecular similarity. The most similar poses are subject to local energy minimization. Both eHits and Surflex use empirical scoring functions but with different terms and parameterization. The molecules studied included TAM and its metabolite 4-hydroxytamoxifen (OHT), raloxifene (RAL), bazedoxifene (BAZ), ormeloxifene (ORM), and lasofoxifene (LAS). Figure 6 depicts their chemical structures. These molecules consist of two moieties: a phenoxy-ethanamine moiety (N-moiety) and a more hydrophobic fragment with two benzene rings (C-moiety). Bonds to break these two moieties are marked by the red bar in Figure 6. Table 1 shows docking scores from eHits. Both of the predicated inhibition sites (TG1 and BHQ) from SERCA are able to bind to TAM and its analogs. However, it is more likely that the TG1 site is the preferred off-target binding site for SERMs because its binding affinity is consistently greater than that of the BHQ site. Analysis of their binding poses when bound to the TG1 site indicates that the N-moiety of these molecules adopt similar binding poses with a specific salt interaction between Glu255 and the amine groups, as shown in Figure 7. This charge neutralization is also observed in ERα when binding to SERMs and is considered the origin of the antiestrogenic effect of SERMs [35]. The binding poses of the C-moiety are more variable due to different conformational constraints. Some of them, such as TAM and BAZ, may have stronger aromatic interactions with the receptor than other SERMs. The predicated binding poses are cross-docked with both eHits 6.2 and Surflex 2.1 and show consistent patterns in binding poses and affinities.

**Table 1:**
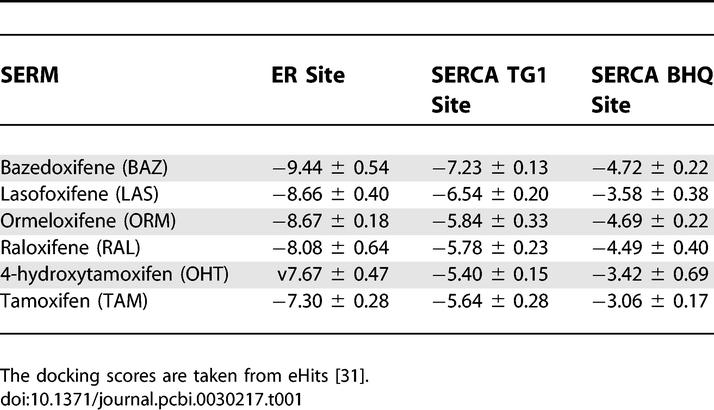
Docking Scores (Unit of log(*K*d)) for the SERM Binding Site to ERα and Predicated Off-Target Binding Sites of SERCA

**Figure 8:**
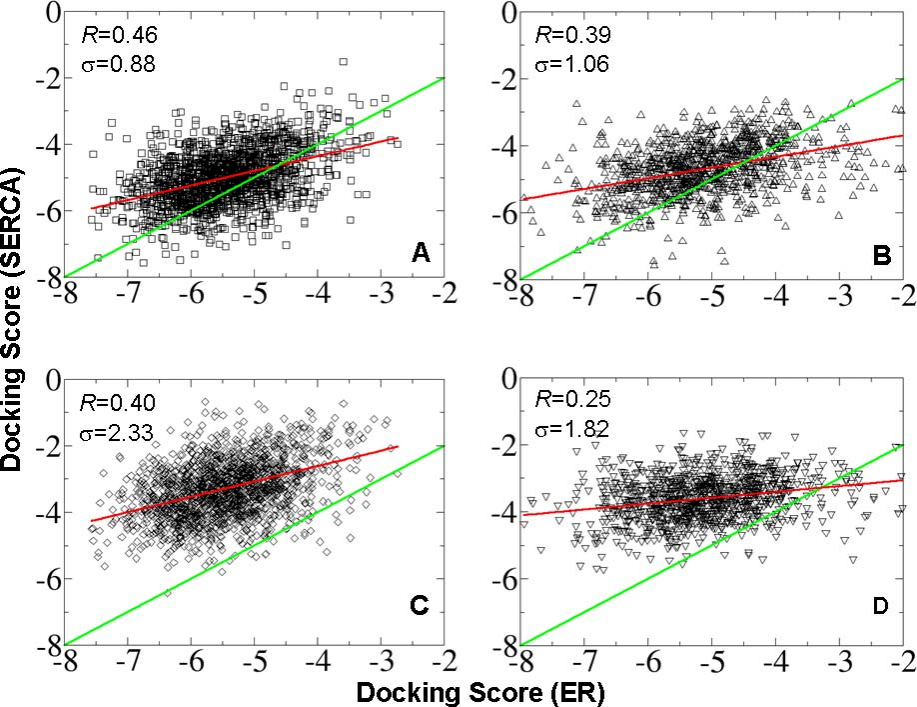
Correlation of Binding Affinity Scores by Docking Molecular Analogs of N-Moiety and C-Moiety of SERMs to ERα and SERCA Proteins (A,B) N- and C-moieties to ERα and SERCA TG1 sites, respectively. (C,D) N- and C-moieties to ERα and SERCA BHQ site, respectively. The red line represents the linear regression of docking scores. The green line indicates the optimal score correlation between two identical binding sites. The docking score is from eHits [31]. Docking score correlations from Surfex [32] show the same trends although the absolute values are different (unpublished data).

Binding affinity alone may not be conclusive because of the poor accuracy of the scoring function [16,36,37]. However, binding poses are able to be predicated reasonably accurately by most docking programs [16,38]. To leverage the strength and weakness of existing docking algorithms, we docked more than 1,000 decoy molecules for both the N-moiety and the C-moiety into the predicated SERCA off-target and primary ERα target sites, respectively. In this way, similar binding sites will show a strong docking score correlation independent of the scoring function, assuming that binding poses are consistent between the two sites. Alternatively, the correlation will be weak if the docking pose is random or the two sites are dissimilar. There were two reasons to break down the molecule into N- and C-moieties. First, the conformational search space of the molecules used in docking will be reduced by using a small fragment and is more likely to predicate their binding pose consistently. Second, the N-moiety has been predicated to have more favorable interactions than the C-moiety. The separate evaluation of their binding affinities will further verify the predicated binding poses and provide insight into designing highly specific SERMs to minimize off-target binding. As shown in Figure 8, the correlation of the eHits docking scores of the N- and C-moiety analogs between the SERM and SERCA TG1 sites is strong relative to that of the BHQ site (Figure 8A and 8C). Taking into account that the correlation between docking scores and experimental affinity is around 0.4–0.6 for most of docking programs [16,36,37], the N-moiety correlation coefficient of 0.46 between SERM and TG1 sites is close to the limit of docking score accuracy. Moreover, the docking score distribution is centered around an optimal correlation line between two identical binding sites (green line in Figure 8) with a standard deviation of 0.88, much less than the 2.33 between the SERM and the BHQ site. Docking score correlations from Surflex show the same pattern as those from eHits. The N-moiety correlation coefficients are 0.50 and 0.44 for the TG1 and BHQ sites, respectively. The corresponding standard deviations are 1.51 and 2.14, respectively. These results are consistent with the predicated binding poses and the relative binding affinities, further supporting the notion that the SERCA TG1 site is similar to the SERM binding site.

There is experimental evidence to support our theoretical off-target binding site. It has been shown that pretreatment with TAM inhibits TG1′s effect in increasing the intracellular Ca2+ concentration [39–41]. One potential mechanism for this observed effect is that TAM binds to the same site as TG1, thus blocking its effect, although it is not clear how TG1 inhibition of SERCA leads to an increase in Ca2+ concentration from these experiments. Our findings indicate that TAM is able to bind directly to the TG1 site and for the first time suggests an inhibition mechanism in atomic detail. Moreover, residue 309 (Glu-309) is included in the BHQ binding sites. It is known that this residue acts as a cytoplasmic gate that allows the release of calcium ions [29]. It is postulated that if TAM interacts with Glu-309 or its interacting partner, it can affect the function of Glu-309 and thus the transport function of the calcium pump as a whole.

Maintaining the level of calcium in the cell is critical to normal cell function. Previous clinical findings have shown that TAM therapy is associated with undesirable side effects such as cardiac abnormalities [19], thromboembolic disorders [20], and ocular toxicity [21]. Recent physiological studies suggest that TAM [42,43], anti-estrogens/β-estradiol [44], phytoestrogens [45], and ovarian sex hormones [46] play important roles in regulating calcium uptake activity of cardiac SR. Given that the gradient concentration of calcium ions in SR is important for muscle contraction [26], it is possible that the cardiac abnormality is caused by its inhibition of SERCA. It has been observed that TAM significantly reduces intracellular calcium concentration and release in the platelets, which is correlated with platelet adhesion and aggregation [47]. The loss of calcium homeostatis in the platelets may originate from inhibition of SERCA by TAM. In addition, there is evidence that diethylstilbestrol increases intracellular calcium in lens epithelial cells by inhibiting SERCA [48] and cataracts result from TG1-inhibited SERCA upregulation [49,50]. We believe the evidence for off-site binding of SERMs to SERCA and the proposed impact that it has on calcium homeostatis leads to the reported adverse effects. As shown in Table 1, in general, the off-target TG1 binding affinity increases with an increase in primary main target binding affinity. However, the binding affinity difference for RAL between target and off-target binding is larger than TAM. Thus, it is expected that RAL exhibits less competitive binding of the SERCA protein, resulting in less adverse effects than TAM. This predication is consistent with clinical studies that show RAL has less adverse effect of thromboembolic disorder and cataract formation than TAM [18]. Among the molecules studied, ORM is predicated as one of the least SERCA competitive binding therapeutics. Clinical studies indeed show that ORM is safe for long-term usage with less adverse effects [51]. LAS and BAZ are newly developed breast cancer therapeutics currently in clinical trial. Although they show the strongest binding to ERα, they also potentially bind strongly to off-targets. It is expected that LAS and BAZ may have similar competitive binding profiles to RAL when binding to SERCA. If indeed this competitive binding and the associated side effects prove to be consistent, the value of this approach to lead optimization and drug development will be further supported.

## Discussion

Adverse effects of clinical drugs begins at the molecular level, involve complex biological networks, and ultimately are measured by clinical outcomes at the level of the whole organism [52]. To correlate protein–drug interactions at the molecule level with their clinical outcomes requires, as a first step, a systemic study of protein–ligand interactions on a proteome-wide scale [5]. Although small molecular similarity alone can provide valuable clues for identifying off-targets [5–12], structural similarity does not always imply the same pattern of biological activity. Conversely, chemicals with dissimilar structures may show the same biological mechanisms of action [53,54]. In principle, the direct assessment of protein–ligand interactions for every ligand and every target in the human proteome will be most valuable for understanding the molecular mechanisms of therapeutic adverse effects by identifying off-target cross-reactivity [13,14]. However, in silico screening through protein–ligand docking is not feasible on a proteome-wide scale. Geometric properties of the protein structure, such as pockets and cavities, and evolutionary linkages between proteins across fold and function space provide rational constraints to address the docking problem [22; Xie and Bourne, submitted]. Thus, identification of similar ligand binding sites significantly reduces the search space that docking needs to address. In this study, we demonstrate the utility of our method to identify the possible mechanism of adverse effect of commercial drugs by combining functional site similarity searching on a structural proteome-wide scale, small molecule screening, and conventional protein–ligand docking. With advances in structural genomics [15,55] and homology modeling [56], it is possible for us to scan a significant and increasing fraction of the whole human proteome to identify for the likely off-targets and to systematically study adverse drug effects at the organism level.

We believe our integrated approach is immediately valuable to the drug discovery and development process. The predicated panel of off-targets can be used to prioritize in vitro screening experiments, thus reducing costs and increasing our ability to identify adverse drug effects. Furthermore, the predicated off-target binding mechanisms provide insights for optimizing drug leads by taking into account not only targeted receptors but also off-targets so that unwanted side effects can be reduced in the early stage of drug development. In our SERM case study, the N-moiety binding sub-sites of ERα and SERCA are more similar than the C-moiety sub-sites. Thus, it is more likely that we will achieve highly specific SERMs by optimizing the C-moiety. In certain cases the primary and off-target binding sites may be highly similar. As a result, it is difficult, if not impossible, to reduce the competitive binding of the off-target by lead optimization. Other strategies have to be applied to minimize the off-target binding; for example, through an increase in bioavailability of drugs, or optimization of the administration regimen. Knowledge of off-targets will be invaluable for this purpose. For instance, SERCA's competitive binding to SERMs can be reduced by delivering SERMs enveloped with hydrophobic agents. SERMs have to first pass through the lipid bilayers of the cytoplasm membrane where the predicated SERMs off-target binding site in SERCA is located. Conceivably, the envelope will reduce off-target binding and permit more of the respective drugs to reach the final ERα target in the nucleus.

### Conclusion

SERMs are potent anti-cancer drugs. By combining, first, functional site similarity searching on a structural proteome-wide scale, second, small molecule screening, and, finally, protein–ligand docking, a potential mechanism for the adverse effect of SERMs has been established. Specifically, we provide evidence for off-target binding of SERMs, resulting in the inhibition of a SERCA transmembrane domain which leads to a disruption in calcium homeostasis. The computational prediction presented here is supported by experimental observations from in vitro and clinical studies. Our methodology provides opportunities to develop further refined SERMs with fewer side effects. On a larger scale there exists the opportunity to explore off-targets binding for any existing pharmaceutical or compound of pharmaceutical interest for which a 3D structural model is available. At this time we are beginning to systematically analyze all commercially available pharmaceuticals in an effort to explain any observed side effects.

## Methods

### Structural models of the human proteome.

Sequences of all PDB [23] structures are mapped to Ensembl [25] human protein sequences (43,738 proteins) using BLAST [24]. A total of 10,730 PDB structures map to 3,158 Ensembl human proteins with a sequence identity above 95%. These 10,730 structures are considered as structural models for the human proteome. They form 2,586 sequence clusters when using a sequence identity of 30%.

### Structural coverage of the druggable human proteome.

The existing druggable human proteome is determined by mapping Ensembl [25] human protein sequences against all sequences of drug targets from Drugbank [57] using BLAST [24]. Homologous sequences from the human proteome, with e-values less than 0.001, constitute the druggable human proteome—a total of 13,865 human proteins corresponding to 2,002 unique drug targets. Among the 10,730 human protein structural models, 1,585 belong to the existing druggable human proteome and correspond to 929 unique drug targets. 825 sequence clusters are formed after clustering the druggable structures with a sequence identity of 30%. One structure is randomly selected from each of the clusters to constitute a representative set of druggable structures. These structures represent approximately 10% of the complete druggable human proteome and 40% of existing drug targets. A flow chart depicting this selection process is given in Figure S3.

### Ligand binding site similarity.

Protein structures are represented by Delaunay tessellation of Cα atoms and characterized with geometric potentials [22]. The similar residue clusters for any protein to a ligand binding site are detected with a SOIPPA algorithm [Xie and Bourne, submitted]. To evaluate the *p*-value for the similarity score calculated from the site comparison method, we estimate the background distribution using a non-parametric method. First, the drug target of interest is compared against the 825 representative sets of human druggable structures. We remove those hits that are in the same fold as the query because they will probably be true positives. Then a kernel density estimator is used to estimate the background probability distribution of the binding site alignment scores. A Gaussian kernel with fixed bandwidth is used. The optimal bandwidth is estimated from the data by using a least square cross-validation approach [58]. Finally, this estimated density function is used to calculate a *p*-value for the particular pair of ligand sites being compared.

### Protein–ligand docking.

Protein–ligand docking is conducted using the eHits [31] and Surflex 2.1 [32] software packages. Default parameter settings were applied when using Surflex. For eHits the accuracy is set to the highest (accuracy = 6) during docking. The highest accuracy means that the most extensive conformational search is performed to determine the ligand binding pose and affinity. Structures of ERα and SERCA proteins were downloaded from the RCSB PDB [23]. The PDB ids of ERα proteins are 1xpc, 3ert, 1r5k, 1err, and 2jfa. The PDB ids of SERCA proteins are 2agv, 1xp5, 1wpg, 1iwo, 2c88, and 2eat.

### Small molecule screening.

**Figure 9:**
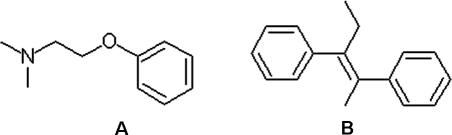
N-Moiety (A) and C-Moiety (B) Molecular Fragments Used in the 2D Graph Similarity Search for Decoy Molecules

The decoy molecules are generated by querying the N- and C-moiety of TAM against the ZINC database [59] using a 2D subgraph similarity search through the associated Web site (http://blaster.docking.org/zinc/). The query molecular structures of the N- and C-moieties are shown in Figure 9. The query generates 1,638 hits to the N-moiety and 1,128 hits to the C-moiety, respectively.

### Electrostatic potential calculation and molecular visualization.

The electrostatic potential of the molecule is calculated using the Gemstone interface (http://gemstone.mozdev.org) to the Adaptive Poisson-Boltzman Solver (APBS) [60]. For calculation of the original ERα drug target (PDB id: 1XPC), the dielectric constant is set to 2.0 for the protein and 78.54 for the solvent. For the off-target SERCA (PDB id: 2AGV), the constant is set to 4.0 for the protein and 78.54 for the solvent, as this molecule is embedded in the phospholipid bilayer. Other parameters are set to defaults as provided by the Gemstone interface. Visualization of the structures is performed using Chimera [61].

## Supporting Information

Figure S1 Background SOIPPA Raw Score Distributions of the Non-Redundant Human 2,586 Set and the Druggable 825 Set When Querying the ERα Ligand Binding Site The distribution of the 2,586 set is slightly shifted to lower scores than that of the 825 set, with means of 44.82 and 48.18, respectively. This is expected because the 2,586 set includes proteins that may not be able to bind drug-like molecules with high affinity.
(1.0 MB TIF)

Figure S2 Alignment of Rabbit and Human SERCA Proteins The transmembrane domain is underlined. The residues centered around TG1 within 10 Å are colored red.
(23 KB DOC)

Figure S3 Flow Chart Depicting the Selection Process of 825 Representative Human Druggable Structural Models (106 KB TIF)

Table S1 PDB Structures Representing the Druggable Human Proteome (130 KB DOC)

Table S2 Top Ten Off-Fold Hits Other Than SERCA Found by Searching the ERα Ligand Binding Site Using SOIPPA Top-ranked proteins that belong to the nuclear receptor fold are not listed because they share the same fold as the template ERα
(1.0 MB TIF)

### Accession Numbers

The protein accession numbers for estrogen receptor alpha are UniProt (http://www.pir.uniprot.org/) P03372 and Protein Data Bank (http://www.rcsb.org/pdb/home/home.do) id 1XPC, and for sarcoplasmic/endoplasmic reticulum calcium ATPase 1 are UniProt P04191 and PDB id 2AGV, 1WPE.

## Abbreviations

BAZ: bazedoxifene
LAS: lasofoxifene
OHT: 4-hydroxytamoxifen
ORM: ormeloxifene
PDB: Protein Data Bank
RAL: raloxifene
SERCA: Sacroplasmic Reticulum Ca2+ ion channel ATPase protein
SERM: selective estrogen receptor modulator
SOIPPA: sequence order independent profile–profile alignment algorithm
TAM: tamoxifen
