# Supplementary figures and images for: In Silico Elucidation of the Molecular Mechanism Defining the Adverse Effect of Selective Estrogen Receptor Modulators
Source: PLoS Comput Biol. 2007 Nov 30;3(11):e217. doi: 10.1371/journal.pcbi.0030217 (PMC2098847; doi:10.1371/journal.pcbi.0030217)

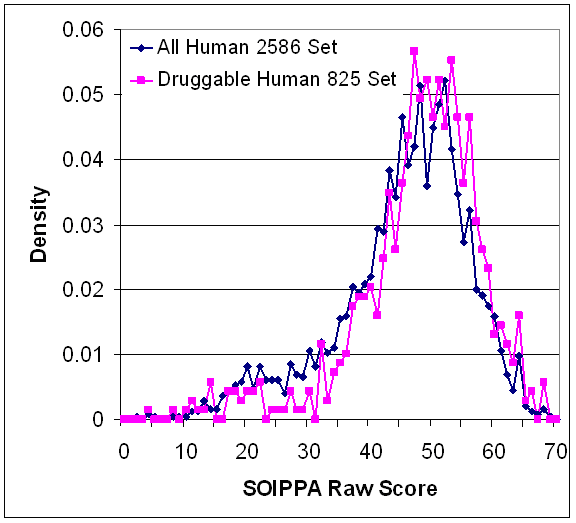

Supplement: Figure S1 — The distribution of the 2,586 set is slightly shifted to lower scores than that of the 825 set, with means of 44.82 and 48.18, respectively. This is expected because the 2,586 set includes proteins that may not be able to bind drug-like molecules with high affinity. (1.0 MB TIF) [file pcbi.0030217.sg001.tif]

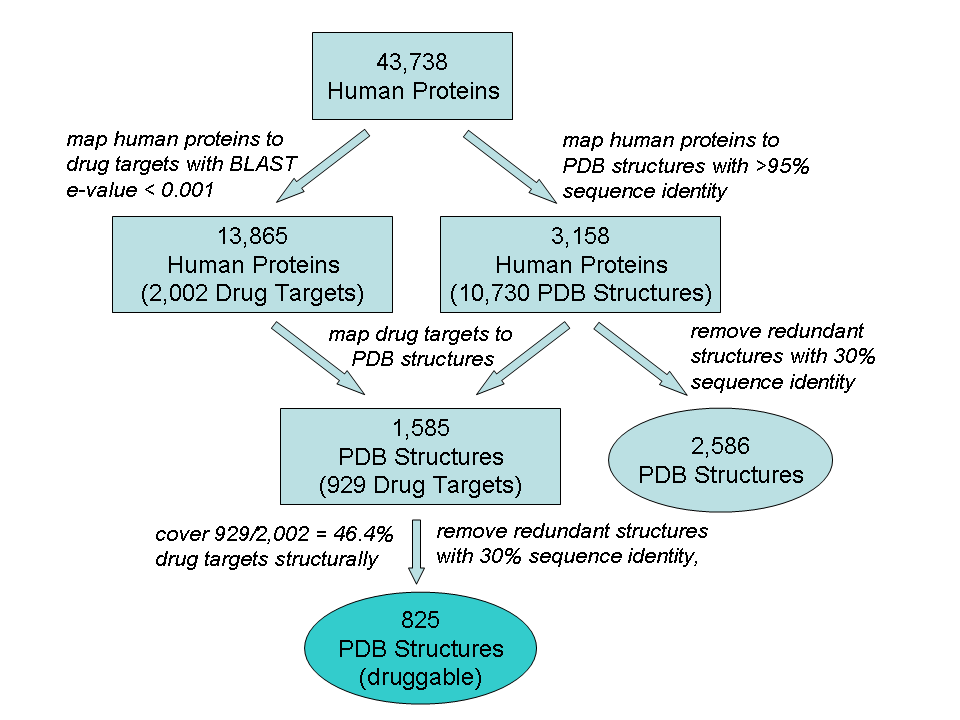

Supplement: Figure S3 — (106 KB TIF) [file pcbi.0030217.sg003.tif]
