# Supplementary material for: In Silico Elucidation of the Molecular Mechanism Defining the Adverse Effect of Selective Estrogen Receptor Modulators
Source: PLoS Comput Biol. 2007 Nov 30;3(11):e217. doi: 10.1371/journal.pcbi.0030217 (PMC2098847; doi:10.1371/journal.pcbi.0030217)
Supplement: Figure S2 — The transmembrane domain is underlined. The residues centered around TG1 within 10 Å are colored red. (23 KB DOC) [file pcbi.0030217.sg002.doc]

Rabbit: 1 MEAAHSKSTEECLAYFGVSETTGLTPDQVKRHLEKYGHNELPAEEGKSLWELVIEQFEDL 60

Human : 1 MEAAHAKTTEECLAYFGVSETTGLTPDQVKRNLEKYGLNELPAEEGKTLWELVIEQFEDL 60

Rabbit: 61 LVRILLLAACISFVLAWFEEGEETITAFVEPFVILLILIANAIVGVWQERNAENAIEALK 120

Human : 61 LVRILLLAACISFVLAWFEEGEETITAFVEPFVILLILIANAIVGVWQERNAENAIEALK 120

Rabbit: 121 EYEPEMGKVYRADRKSVQRIKARDIVPGDIVEVAVGDKVPADIRILSIKSTTLRVDQSIL 180

Human : 121 EYEPEMGKVYRADRKSVQRIKARDIVPGDIVEVAVGDKVPADIRILAIKSTTLRVDQSIL 180

Rabbit: 181 TGESVSVIKHTEPVPDPRAVNQDKKNMLFSGTNIAAGKALGIVATTGVSTEIGKIRDQMA 240

Human : 181 TGESVSVIKHTEPVPDPRAVNQDKKNMLFSGTNIAAGKALGIVATTGVGTEIGKIRDQMA 240

Rabbit: 241 ATEQDKTPLQQKLDEFGEQLSKVISLICVAVWLINIGHFNDPVHGGSWIRGAIYYFKIAV 300

Human : 241 ATEQDKTPLQQKLDEFGEQLSKVISLICVAVWLINIGHFNDPVHGGSWFRGAIYYFKIAV 300

Rabbit: 301 ALAVAAIPEGLPAVITTCLALGTRRMAKKNAIVRSLPSVETLGCTSVICSDKTGTLTTNQ 360

Human : 301 ALAVAAIPEGLPAVITTCLALGTRRMAKKNAIVRSLPSVETLGCTSVICSDKTGTLTTNQ 360

Rabbit: 361 MSVCKMFIIDKVDGDFCSLNEFSITGSTYAPEGEVLKNDKPIRSGQFDGLVELATICALC 420

Human : 361 MSVCKMFIIDKVDGDICLLNEFSITGSTYAPEGEVLKNDKPVRPGQYDGLVELATICALC 420

Rabbit: 421 NDSSLDFNETKGVYEKVGEATETALTTLVEKMNVFNTEVRNLSKVERANACNSVIRQLMK 480

Human : 421 NDSSLDFNEAKGVYEKVGEATETALTTLVEKMNVFNTDVRSLSKVERANACNSVIRQLMK 480

Rabbit: 481 KEFTLEFSRDRKSMSVYCSPAKSSRAAVGNKMFVKGAPEGVIDRCNYVRVGTTRVPMTGP 540

Human : 481 KEFTLEFSRDRKSMSVYCSPAKSSRAAVGNKMFVKGAPEGVIDRCNYVRVGTTRVPLTGP 540

Rabbit: 541 VKEKILSVIKEWGTGRDTLRCLALATRDTPPKREEMVLDDSSRFMEYETDLTFVGVVGML 600

Human : 541 VKEKIMAVIKEWGTGRDTLRCLALATRDTPPKREEMVLDDSARFLEYETDLTFVGVVGML 600

Rabbit: 601 DPPRKEVMGSIQLCRDAGIRVIMITGDNKGTAIAICRRIGIFGENEEVADRAYTGREFDD 660

Human : 601 DPPRKEVTGSIQLCRDAGIRVIMITGDNKGTAIAICRRIGIFGENEEVADRAYTGREFDD 660

Rabbit: 661 LPLAEQREACRRACCFARVEPSHKSKIVEYLQSYDEITAMTGDGVNDAPALKKAEIGIAM 720

Human : 661 LPLAEQREACRRACCFARVEPSHKSKIVEYLQSYDEITAMTGDGVNDAPALKKAEIGIAM 720

Rabbit: 721 GSGTAVAKTASEMVLADDNFSTIVAAVEEGRAIYNNMKQFIRYLISSNVGEVVCIFLTAA 780

Human : 721 GSGTAVAKTASEMVLADDNFSTIVAAVEEGRAIYNNMKQFIRYLISSNVGEVVCIFLTAA 780

Rabbit: 781 LGLPEALIPVQLLWVNLVTDGLPATALGFNPPDLDIMDRPPRSPKEPLISGWLFFRYMAI 840

Human : 781 LGLPEALIPVQLLWVNLVTDGLPATALGFNPPDLDIMDRPPRSPKEPLISGWLFFRYMAI 840

Rabbit: 841 GGYVGAATVGAAAWWFMYAEDGPGVTYHQLTHFMQCTEDHPHFEGLDCEIFEAPEPMTMA 900

Human : 841 GGYVGAATVGAAAWWFLYAEDGPHVNYSQLTHFMQCTEDNTHFEGIDCEVFEAPEPMTMA 900

Rabbit: 901 LSVLVTIEMCNALNSLSENQSLMRMPPWVNIWLLGSICLSMSLHFLILYVDPLPMIFKLK 960

Human : 901 LSVLVTIEMCNALNSLSENQSLLRMPPWVNIWLLGSICLSMSLHFLILYVDPLPMIFKLR 960

Rabbit: 961 ALDLTQWLMVLKISLPVIGLDEILKFIARNYLE 993

Human : 961 ALDLTQWLMVLKISLPVIGLDEILKFVARNYLE 993

**Figure 2.** Alignment of rabbit and human SERCA protein. Transmembrane domain is underscored. The residues centered around TG1 within 10 Å are colored as red.
