# Supplementary material for: In Silico Elucidation of the Molecular Mechanism Defining the Adverse Effect of Selective Estrogen Receptor Modulators
Source: PLoS Comput Biol. 2007 Nov 30;3(11):e217. doi: 10.1371/journal.pcbi.0030217 (PMC2098847; doi:10.1371/journal.pcbi.0030217)
Supplement: Table S1 — (130 KB DOC) [file pcbi.0030217.st001.doc]

**Table S1. Representative PDB structures for druggable human proteome**

**PDB ENSEMBL ID Drug Target**

2CFI ENSP00000273450 10-Formyltetrahydrofolate Dehydrogenase - Rattus

norvegicus (Rat)

2BR9 ENSP00000264335 14-3-3-Like Protein C - Nicotiana tabacum (Common

tobacco)

1YB1 ENSP00000351035 17-Beta-Hydroxysteroid Dehydrogenase Type Xi - Homo

sapiens

1SMB ENSP00000367196 17Kd Fetal Brain Protein - T4-like viruses

Bacteriophage T4

1W8D ENSP00000220764 2,4-Dienoyl-Coa Reductase - Rana perezi (Perez's

frog)

2GTR ENSP00000330512 2-Enoyl-Coa Hydratase - Rattus norvegicus (Rat)

1Z1L ENSP00000334910 3',5'-Cyclic Nucleotide Phosphodiesterase 2A - Mus

musculus (Mouse)

1ZBU ENSP00000250263 3'-5' Exonuclease Eri1 - Mycobacterium tuberculosis

1SG4 ENSP00000301729 3,2-Trans-Enoyl-Coa Isomerase,

2J91 ENSP00000216194 3-Carboxy-Cis, Cis-Muconate Cycloisomerase –

Pyrococcus furiosus

2P8U ENSP00000322706 3-Hydroxy-3-Methylglutaryl-Coa Synthase –

Staphylococcus aureus

2IIK ENSP00000333664 3-Ketoacetyl-Coa Thiolase - Methanococcus jannaschii

2JFK ENSP00000363777 3-Oxoacyl- - Thermus thermophilus

2PE2 ENSP00000371763 3-Phosphoinositide Dependent Protein Kin –

Methanococcus jannaschii

2O23 ENSP00000168216 3-hydroxyacyl-CoA dehydrogenase type II

2I9P ENSP00000265395 3-hydroxyisobutyrate dehydrogenase, mitochondrial

precursor

2DZJ ENSP00000215567 3-oxo-5-alpha-steroid 4-dehydrogenase 2

2CG5 ENSP00000278618 4'-Phosphopantetheinyl Transferase Sfp - Escherichia

coli

2CQU ENSP00000369461 4-Chlorobenzoyl Coenzyme A Dehalogenase - Pseudomonas

sp.

2F15 ENSP00000254101 5'-AMP-activated protein kinase, beta-2 subunit

2YWP ENSP00000278916 5'-AMP-activated protein kinase, catalytic alpha-1

chain

1SD2 ENSP00000369519 5'-Deoxy-5'-Methylthioadenosine Phosphorylas - Homo

sapiens (Human)

1Q92 ENSP00000373675 5 - Homo sapiens (Human)

2I1V ENSP00000369100 6-Phosphofructo-2-Kinase/ Fructose-2,6- Bisp –

Thermus thermophilus

2DIG ENSP00000272163 7-dehydrocholesterol reductase

7HSC ENSP00000227378 70 Kilodalton Heat Shock Protein - Mus musculus

(Mouse)

1WXM ENSP00000366238 A-Raf proto-oncogene serine/threonine-protein kinase

1X51 ENSP00000361187 A/G-Specific Adenine Glycosylase - Thermotoga

maritima

1QVJ ENSP00000303575 ADP-Ribose Pyrophosphatase - Bos taurus (Bovine)

2DUK ENSP00000338352 ADP-Ribose Pyrophosphatase - Thermus thermophilus

2B6H ENSP00000000233 ADP-Ribosylation Factor 4 - Triticum aestivum (Wheat)

2V1X ENSP00000318727 ATP-Dependent DNA Helicase

1VEC ENSP00000264018 ATP-Dependent RNA Helicase P54 - Escherichia coli

2IBY ENSP00000265838 Acetyl-CoA Acetyltransferase (Mitochondrial)

1WL5 ENSP00000356015 Acetyl-Coenzyme A Acetyltransferase 2 - Escherichia

coli

1VZJ ENSP00000241069 Acetylcholinesterase (AChE)

2B3Y ENSP00000369255 Aconitase - Sus scrofa (Pig)

2P9U ENSP00000263238 Actin, Alpha Skeletal Muscle - Rattus norvegicus

(Rat)

1Q8X ENSP00000374299 Actin Depolymerizing Factor - Escherichia coli

1U54 ENSP00000323216 Activated CDC42 kinase 1

2HDX ENSP00000371067 Activated Cdc42 Kinase 1 - Homo sapiens (Human)

2JIF ENSP00000357873 Acyl-CoA dehydrogenase

2FJ9 ENSP00000348116 Acyl-Coenzyme A Binding Protein - Escherichia coli

2I47 ENSP00000339991 Adam 33 - Trichoderma reesei (Hypocrea jecorina)

1ZN9 ENSP00000373425 Adenine Phosphoribosyltransferase - Mycobacterium

tuberculosis

2C9Y ENSP00000346921 Adenylate Kinase - Bacillus subtilis

2C95 ENSP00000362271 Adenylate Kinase 1 - Bacillus subtilis

1ZD8 ENSP00000371230 Adenylate Kinase Isoenzyme-3 - Mycobacterium

tuberculosis

2V40 ENSP00000355493 Adenylosuccinate Synthetase - Mus musculus (Mouse)

2ALR ENSP00000361140 Alcohol dehydrogenase [NADP+]

1U3T ENSP00000209668 Alcohol dehydrogenase alpha chain

1D1S ENSP00000209665 Alcohol dehydrogenase class IV mu/sigma chain

1OF7 ENSP00000261733 Aldehyde dehydrogenase, mitochondrial precursor

2J6L ENSP00000297542 Aldehyde dehydrogenase family 7 member A1

2FVL ENSP00000369814 Aldo-keto reductase family 1 member C4

1XFB ENSP00000226253 Aldolase - Oryctolagus cuniculus (Rabbit)

1SO0 ENSP00000368183 Aldose 1-Epimerase - Lactococcus lactis

1TR2 ENSP00000361841 Alpha-1 Catenin - Methanococcus jannaschii

1HOF ENSP00000358446 Alpha-2A Adrenergic Receptor

2DH3 ENSP00000367123 Alpha-Amylase II - Thermoactinomyces vulgaris

1R47 ENSP00000218516 Alpha-Galactosidase A - Mus musculus (Mouse)

1R5L ENSP00000260116 Alpha-Tocopherol Transfer Protein - Homo sapiens

(Human)

1I5H ENSP00000345751 Amiloride-sensitive sodium channel beta-subunit

1Q7L ENSP00000232907 Aminoacylase-1

1WSV ENSP00000273588 Aminomethyltransferase

2G47 ENSP00000284981 Amyloid Beta-Peptide - Arabidopsis thaliana (Mouse-

ear cress)

2PX9 ENSP00000246548 Amyloid protein-binding protein 1

2Q7K ENSP00000363822 Androgen receptor

2AJF ENSP00000369713 Angiotensin-converting enzyme (ACE)

2HYW ENSP00000346032 Annexin A1

1M9I ENSP00000346550 Annexin V - Pan troglodytes (Chimpanzee)

1W45 ENSP00000363395 Annexin V - Rattus norvegicus (Rat)

1TZN ENSP00000306185 Anthrax Toxin Receptor 2 - Shigella sonnei

2HLR ENSP00000373711 Anti-Muellerian hormone type II receptor

2HIJ ENSP00000356671 Antithrombin III (ATIII)

2OU1 ENSP00000356969 Apolipoprotein A-II - Thermus thermophilus

2PQK ENSP00000358022 Apoptosis regulator Bcl-2

3YGS ENSP00000353059 Apoptotic Protease Activating Factor 1 - Mus musculus

(Mouse)

2H6Q ENSP00000351446 Apoptotic protease activating factor 1

2H2N ENSP00000307674 Apyrase - Shigella sonnei

1IH5 ENSP00000311165 Aquaporin 1 - Bos taurus (Bovine)

2PHO ENSP00000357066 Arginase I

1PQ3 ENSP00000261783 Arginase II

1K62 ENSP00000307188 Arginosuccinate lyase

2NZ2 ENSP00000253004 Arginosuccinate synthase

1PBV ENSP00000314566 Arno - Escherichia coli

1XG5 ENSP00000251312 Arpg836 - Homo sapiens

2PFR ENSP00000286479 Arylamine N-Acetyltransferase - Mycobacterium

smegmatis

1N2L ENSP00000348406 Arylsulfatase A - Homo sapiens (Human)

2Q51 ENSP00000371361 Aspartoacylase

1YK1 ENSP00000265074 Atrial Natriuretic Peptide Clearance Recepto - Homo

sapiens (Human)

2OJX ENSP00000300093 Aurora-Related Kinase 1 - Mus musculus (Mouse)

2OWI ENSP00000356430 Axin - Bacillus subtilis

2OID ENSP00000349096 B-Raf proto-oncogene serine/threonine-protein kinase

1OTZ ENSP00000365048 B Lymphocyte Stimulator - Pyrococcus furiosus

1EWF ENSP00000262865 Bactericidal/Permeability-Increasing Protein - Homo

sapiens (Human)

1XOX ENSP00000324180 Baculoviral Iap Repeat-Containing Protein 4 -

1XB1 ENSP00000321377 Baculoviral Iap Repeat-Containing Protein 4 - Homo

sapiens (Human)

4FGF ENSP00000264498 Basic Fibroblast Growth Factor - Rattus norvegicus

(Rat)

1YTQ ENSP00000215861 Beta-B2-Crystallin - Chain - Rattus norvegicus

(Rat)

3BCT ENSP00000344456 Beta-Catenin - Escherichia coli

2JFE ENSP00000314501 Beta-Glucosidase - Thermotoga maritima

1BHG ENSP00000302728 Beta-Glucuronidase - Trichoderma reesei (Hypocrea

jecorina)

2GK1 ENSP00000268097 Beta-Hexosaminidase Beta Chain - Thermotoga maritima

1XKI ENSP00000263598 Beta-Lactoglobulin - Mus musculus (Mouse)

2P83 ENSP00000318585 Beta-Secretase 1 - Homo sapiens (Human)

2J59 ENSP00000365604 Beta-Spectrin - Rattus norvegicus (Rat)

2GDD ENSP00000372254 Beta-Tryptase - Homo sapiens (Human)

1BAK ENSP00000312262 Beta-adrenergic receptor kinase 1

2ACX ENSP00000347655 Beta-adrenergic receptor kinase 2

1C1Z ENSP00000205948 Beta2-Glycoprotein-I - Trichoderma reesei (Hypocrea

jecorina)

1OKI ENSP00000215939 Beta Crystallin B1 - Staphylococcus aureus

2PLE ENSP00000261799 Beta platelet-derived growth factor precursor (PDGF-

R-beta, CD140b)

2OFX ENSP00000265174 Bifunctional 3'-Phosphoadenosine 5'- Phospho - Mus

musculus (Mouse)

1PL0 ENSP00000236959 Bifunctional Purine Biosynthesis Protein Pur - Homo

sapiens (Human)

1BIK ENSP00000265132 Bikunin

1OSH ENSP00000188403 Bile Acid Receptor - Homo sapiens (Human)

1MVU ENSP00000265723 Bile salt export pump

2H63 ENSP00000265523 Biliverdin reductase A precursor

1SRA ENSP00000231061 Bm-40

2EF1 ENSP00000371779 Bone Marrow Stromal Cell Antigen 1 - Archaeoglobus

fulgidus

2P6A ENSP00000242208 Bone Morphogenetic Protein 2 - Methanococcus jannaschii

1M4U ENSP00000360340 Bone Morphogenetic Protein 7

2COJ ENSP00000261192 Branched-chain-amino-acid aminotransferase, cytosolic

2HHF ENSP00000322991 Branched-chain-amino-acid aminotransferase,

mitochondrial

2UXW ENSP00000349297 Butyryl-Coa Dehydrogenase - Rattus norvegicus (Rat)

1DIG ENSP00000216605 C-1-tetrahydrofolate synthase

2FN4 ENSP00000246792 C-Ha-Ras - Triticum aestivum (Wheat)

1LJ7 ENSP00000255030 C-Reactive Protein - Bos taurus (Bovine)

2OME ENSP00000357816 C-Terminal Binding Protein 3 -

1JWO ENSP00000308734 C-Terminal Src Kinase - Homo sapiens (Human)

2C6U ENSP00000298527 C-Type Lectin Dc-Signr - Homo sapiens (Human)

2OSL ENSP00000374591 CD20 B-lymphocyte antigen

1HNF ENSP00000358490 CD2 lymphocyte antigen

2HRL ENSP00000323328 CD33 antigen

2GK2 ENSP00000341688 CEA or carcinoembryonic antigen-multiple sequences

2AVD ENSP00000361616 Caffeoyl-Coa O-Methyltransferase - Medicago sativa

(Alfalfa)

2P6B ENSP00000367201 Calcineurin B subunit isoform 1

1KFX ENSP00000246533 Calcium-Dependent Protease, Small Subunit - Sus

scrofa (Pig)

2ARF ENSP00000242839 Calcium-transporting ATPase type 2C member 1

1TAZ ENSP00000243052 Calcium/Calmodulin-Dependent 3',5'-Cycli –

Escherichia coli

1HKX ENSP00000305090 Calcium/Calmodulin-Dependent Protein Kinase –

Bacillus subtilis

1A2X ENSP00000361636 Calmodulin

2NUZ ENSP00000361824 Calmodulin - Saccharomyces cerevisiae (Baker's yeast)

2GGM ENSP00000359300 Calmodulin - Shigella sonnei

2QFE ENSP00000253693 Calpain 1, Large [Catalytic] Subunit - Rattus

norvegicus (Rat)

2UZW ENSP00000309591 Camp-Dependent Protein Kinase, Alpha-Cat –

Methanococcus jannaschii

1ZXA ENSP00000327642 Camp-Dependent Protein Kinase, Alpha-Cat - Mus

musculus (Mouse)

1V9V ENSP00000262811 Camp-Dependent Protein Kinase - Mus musculus (Mouse)

2IZX ENSP00000265563 Camp-Dependent Protein Kinase Regulatory Sub -

1RL3 ENSP00000351410 Camp Dependent Protein Kinase - Rattus norvegicus

(Rat)

2NN7 ENSP00000256119 Carbonic Anhydrase I

1ZNC ENSP00000300900 Carbonic Anhydrase IV

1JD0 ENSP00000178638 Carbonic Anhydrase Xii -

1WMA ENSP00000290349 Carbonyl Reductase [Nadph] 1 - Homo sapiens (Human)

2Q5E ENSP00000309934 Carboxy-Terminal Domain RNA Polymerase I - Mus

musculus (Mouse)

1FJ2 ENSP00000320043 Carboxylesterase - Bacillus subtilis

2PCU ENSP00000222482 Carboxypeptidase A - Bos taurus (Bovine)

2NSM ENSP00000359446 Carboxypeptidase Gp180 Residues 503-882 -

1UWY ENSP00000339157 Carboxypeptidase M

2IZU ENSP00000353904 Casein Kinase-1 - Schizosaccharomyces pombe (Fission

yeast)

2PVR ENSP00000371400 Casein Kinase II, Alpha Chain - Escherichia coli

2HBY ENSP00000350493 Caspase-1 precursor

2J30 ENSP00000311032 Caspase-3 - Homo sapiens (Human)

2P2C ENSP00000312664 Caspase-7 - Homo sapiens (Human)

1QQW ENSP00000241052 Catalase - Pseudomonas stutzeri (Pseudomonas

perfectomarina)

3PBH ENSP00000345672 Cathepsin B - Homo sapiens (Human)

7PCK ENSP00000271651 Cathepsin K - Macaca mulatta (Rhesus macaque)

2OP3 ENSP00000357981 Cathepsin S - Homo sapiens (Human)

1JUQ ENSP00000000412 Cation-Dependent Mannose-6-Phosphate Recepto

1LF8 ENSP00000349437 Cation-Independent Mannose 6-Phosphate Recep - Bos

taurus (Bovine)

2O39 ENSP00000314664 Cd46 - Trichoderma reesei (Hypocrea jecorina)

1ERH ENSP00000340210 Cd59 Complexed With Glcnac-Beta-1,

2Q3H ENSP00000355652 Cdc42Hs-GDP - Triticum aestivum (Wheat)

2CCH ENSP00000209728 Cell Division Control Protein 6 - Pyrobaculum

aerophilum

2V0D ENSP00000266970 Cell Division Protein Kinase 2 - Homo sapiens (Human)

1UA2 ENSP00000370212 Cell Division Protein Kinase 7 - Mus musculus (Mouse)

3CBS ENSP00000357205 Cellular retinoic acid binding protein 2

1SOJ ENSP00000282096 Cgmp-Inhibited 3',5'-Cyclic Phosphodiesteras - Homo

sapiens (Human)

2HD1 ENSP00000291539 Cgmp Phosphodiesterase A2 - Homo sapiens

1NWU ENSP00000255409 Chitinase-3 Like Protein 1

1WB0 ENSP00000356198 Chitotriosidase 1 - Homo sapiens (Human)

2D2Z ENSP00000363500 Chloride Intracellular Channel Protein 1

2PFI ENSP00000332771 Chloride channel protein 2

1L4T ENSP00000335544 Cholecystokinin type A receptor

1PEI ENSP00000292823 Choline-phosphate cytidylyltransferase A

2I7Q ENSP00000265689 Choline kinase alpha

2J4C ENSP00000264381 Cholinesterase

1XWD ENSP00000358595 Chorionic Gonadotropin - Trichoderma reesei (Hypocrea

jecorina)

1NN6 ENSP00000250378 Chymase

1RFN ENSP00000218099 Coagulation Factor IX

1LJ5 ENSP00000223095 Coagulation Factor VIII

2UWP ENSP00000364709 Coagulation Factor X

2PUQ ENSP00000329546 Coagulation factor VII

1QRK ENSP00000264870 Coagulation factor XIII A chain precursor

1R4X ENSP00000325002 Coatomer Gamma Subunit - Homo sapiens (Human)

2IDX ENSP00000266839 Cob(I)yrinic acid a,c-diamide adenosyltransferase

2OX8 ENSP00000344706 Collagen

830C ENSP00000260302 Collagenase 3 - Homo sapiens (Human)

1ELV ENSP00000328173 Complement C1S Component - Homo sapiens (Human)

1KJS ENSP00000223642 Complement C3Dg - Mus musculus (Mouse)

1OK9 ENSP00000316333 Complement Decay-Accelerating Factor - Shigella

sonnei

2ODQ ENSP00000299367 Complement Factor B - Homo sapiens (Human)

3IL8 ENSP00000306512 Connective Tissue Activating Peptide-III - Homo

sapiens (Human)

1XVP ENSP00000356961 Constitutive Androstane Receptor - Mus musculus

(Mouse)

1TL5 ENSP00000316854 Copper Transport Protein Atox1 - Pseudomonas

aeruginosa

2IRW ENSP00000355995 Corticosteroid 11-beta-dehydrogenase, isozyme 1

1N9U ENSP00000355627 Corticosteroid Receptor

2GL6 ENSP00000312588 Creatine kinase, sarcomeric

1I0E ENSP00000221476 Creatine kinase M-type

2D1X ENSP00000301843 Crk-Associated Substrate - Mus musculus (Mouse)

1YJD ENSP00000324890 Ctla-4 - Homo sapiens (Human)

2C5M ENSP00000361704 Ctp Synthase - Methanococcus jannaschii

1ZWG ENSP00000282091 Cyclic Parathyroid Hormone - Homo sapiens

2AST ENSP00000344538 Cyclin Dependent Kinase Subunit, Type 1 - Mus

musculus (Mouse)

1M54 ENSP00000352643 Cystathionine beta-synthase

2NMP ENSP00000359976 Cystathionine gamma-lyase

2HDY ENSP00000254663 Cysteine desulfurase

2IC1 ENSP00000250535 Cysteine dioxygenase type I

1NRR ENSP00000321326 Cysteinyl leukotriene receptor 2

1CKY ENSP00000003084 Cystic Fibrosis Transmembrane Conductance Re -

1J3S ENSP00000307786 Cytochrome C - Pseudomonas aeruginosa

2HI4 ENSP00000342007 Cytochrome P450 17A1

1R9O ENSP00000260682 Cytochrome P450 2C9 -

2V0M ENSP00000337915 Cytochrome P450 3A4 -

2DC3 ENSP00000293230 Cytoglobin - Pseudomonas stutzeri (Pseudomonas

perfectomarina)

1EGJ ENSP00000262825 Cytokine Receptor Common Beta Chain - Homo sapiens

(Human)

1BCI ENSP00000356436 Cytosolic phospholipase A2

2HLB ENSP00000354859 D(2) Dopamine Receptor

2G76 ENSP00000358417 D-3-phosphoglycerate dehydrogenase

2GSE ENSP00000369966 D-Hydantoinase - Burkholderia pickettii (Pseudomonas

pickettii)

2E82 ENSP00000228476 D-amino-acid oxidase

2JM1 ENSP00000362441 DNA Helicase

1X9N ENSP00000263274 DNA Ligase I

2E2W ENSP00000310449 DNA Ligase III

9ICY ENSP00000265421 DNA Polymerase (human)

1N5G ENSP00000368349 DNA Polymerase alpha (human)

1FU1 ENSP00000282268 DNA Repair Protein Xrcc4 -

1V5W ENSP00000216024 DNA Repair and Recombination Protein Rad - Homo

sapiens (Human)

1TL8 ENSP00000354522 DNA Topoisomerase I

1F93 ENSP00000299299 Dcoh - Homo sapiens (Human)

2JC6 ENSP00000368124 Death-Associated Protein Kinase - Escherichia coli

2JAM ENSP00000354861 Death-Associated Protein Kinase 1 - Homo sapiens

(Human)

1E51 ENSP00000363290 Delta-aminolevulinic acid dehydratase

2OCP ENSP00000264093 Deoxycytidine Kinase - Mus musculus (Mouse)

1RQD ENSP00000210060 Deoxyhypusine Synthase - Homo sapiens (Human)

1R79 ENSP00000264057 Diacylglycerol kinase

2FVV ENSP00000351650 Diadenosine Tetraphosphate Hydrolase - Mycobacterium

tuberculosis

2JEV ENSP00000368572 Diamine acetyltransferase 1

2DHF ENSP00000343033 Dihydrofolate Reductase

2FQI ENSP00000219240 Dihydroorotate Dehydrogenase, mitochondrial

[Precursor]

1HDR ENSP00000371717 Dihydropteridine Reductase

1XFD ENSP00000252510 Dipeptidyl Aminopeptidase-Like Protein 6 -

2OQV ENSP00000353731 Dipeptidyl Peptidase IV - Homo sapiens (Human)

2DJG ENSP00000227266 Dipeptidyl Peptidase I

1FB8 ENSP00000296414 Dual Adaptor Of Phosphotyrosine and 3- Phosp - Homo

sapiens (Human)

2O2V ENSP00000346493 Dual Specificity Mitogen-Activated Protein K - Homo

sapiens (Human)

2EXE ENSP00000328452 Dual Specificity Protein Kinase Clk1 - Homo sapiens

(Human)

2HQU ENSP00000249783 Dutp Pyrophosphatase - Homo sapiens (Human)

1G1T ENSP00000356755 E-Selectin - Homo sapiens (Human)

1X4U ENSP00000359646 Early Endosomal Autoantigen 1 - Homo sapiens (Human)

1FMI ENSP00000360645 Endoplasmic Reticulum Mannosyl-Oligosacchari - Homo

sapiens (Human)

2HG1 ENSP00000299767 Endoplasmin - Canis familiaris (Dog)

2COC ENSP00000364631 Endosome-Associated Protein - Homo sapiens (Human)

1LQV ENSP00000363601 Endothelial Protein C Receptor

1V6R ENSP00000368683 Endothelin-1 - Synthetic construct

1Y8J ENSP00000353679 Enkephalinase

2AKZ ENSP00000229277 Enolase - Saccharomyces cerevisiae (Baker's yeast)

1HZD ENSP00000364883 Enoyl-Coa Hydratase - Rattus norvegicus (Rat)

1K2A ENSP00000303276 Eosinophil-Derived Neurotoxin - Homo sapiens (Human)

1QKQ ENSP00000221804 Eosinophil Lysophospholipase Chain: A - Bos taurus

(Bovine)

2A4C ENSP00000268602 Ep-Cadherin

1SHX ENSP00000369072 Ephrin-A5

2AHX ENSP00000342235 Epidermal Growth Factor Receptor

1ZD5 ENSP00000315621 Epoxide Hydrolase 2, Cytoplasmic - Homo sapiens

(Human)

1XGW ENSP00000296951 Epsin - Rattus norvegicus (Rat)

1ZQ9 ENSP00000199320 Ermc' Methyltransferase - Shigella sonnei

2JIX ENSP00000222139 Erythropoietin receptor

2OZU ENSP00000265713 Esa1 Histone Acetyltransferase - Escherichia coli

1A27 ENSP00000225929 Estradiol 17 Beta-Dehydrogenase 1 - Homo sapiens

(Human)

2PJL ENSP00000000442 Estrogen-Related Receptor Gamma - Homo sapiens

(Human)

2GPV ENSP00000355907 Estrogen-Related Receptor Gamma - Rattus norvegicus

(Rat)

3ERT ENSP00000206249 Estrogen Receptor (ER)

2GIU ENSP00000343925 Estrogen receptor

2JGC ENSP00000258416 Eukaryotic Translation Initiation Factor 4E - Homo

sapiens (Human)

2I6L ENSP00000261845 Extracellular Signal-Regulated Kinase 2 - Mus

musculus (Mouse)

2PMW ENSP00000303208 Extracellular Subtilisin-Like Serine Protein - Vibrio

sp. pa-44

4FAP ENSP00000354587 FKBP12-rapamycin complex-associated protein

1MZD ENSP00000370807 Factor D - Homo sapiens (Human)

2CGO ENSP00000299163 Factor Inhibiting Hif1 - Streptomyces clavuligerus

2F9K ENSP00000357340 Farnesyl Pyrophosphate Synthetase - Escherichia coli

1JJJ ENSP00000300149 Fatty Acid Binding Protein - Mus musculus (Mouse)

2FCB ENSP00000351497 Fc Gamma Receptor FCGR1 HUMAN

2FHA ENSP00000273550 Ferritin heavy chain

2FG8 ENSP00000366525 Ferritin light chain

2HRE ENSP00000372326 Ferrochelatase - Homo sapiens (Human)

3FIB ENSP00000336829 Fibrin

2FDB ENSP00000321797 Fibroblast Growth Factor 9 - Homo sapiens (Human)

2P4I ENSP00000369379 Fibroblast Growth Factor Receptor 2 - Homo sapiens

(Human)

2P39 ENSP00000237837 Fibroblast growth factor-4 precursor

2F2D ENSP00000222308 Fk506-Binding Protein 4 - Homo sapiens (Human)

2PBC ENSP00000310935 Fk506 Binding Protein - Oryctolagus cuniculus

(Rabbit)

1C9H ENSP00000370373 Fkbp12.6 - Oryctolagus cuniculus (Rabbit)

1PBK ENSP00000216330 Fkbp25 - Oryctolagus cuniculus (Rabbit)

1NUU ENSP00000296202 Fksg76 - Streptomyces clavuligerus

1HE5 ENSP00000373547 Flavin reductase

2ETM ENSP00000341189 Focal Adhesion Kinase 1 - Mus musculus (Mouse)

6FIT ENSP00000342087 Fragile Histidine Triad Protein - Homo sapiens

(Human)

2FIX ENSP00000364475 Fructose-1,6-Bisphosphatase - Mycobacterium

tuberculosis

1Q69 ENSP00000283635 Fv Fragment - Mus musculus

1T2A ENSP00000370194 GDP-Mannose 4,6 Dehydratase - Triticum aestivum

(Wheat)

1MOT ENSP00000361700 GLycine alpha 2 receptor

1FB1 ENSP00000254299 GTP Cyclohydrolase I - Homo sapiens (Human)

2A2D ENSP00000316632 Galactokinase - Escherichia coli

2D0J ENSP00000230053 Galactosylgalactosylxylosylprotein 3-Beta- G

1GZW ENSP00000215909 Galectin-1 - Trichoderma reesei (Hypocrea jecorina)

2NN8 ENSP00000254301 Galectin-3 - Homo sapiens (Human)

1L9X ENSP00000260118 Gamma-glutamyl hydrolase

1PUB ENSP00000349687 Ganglioside Gm2 Activator - Shigella sonnei

2GH0 ENSP00000274721 Gdnf Family Receptor Alpha 1 - Methanococcus

jannaschii

2FU3 ENSP00000303019 Gephyrin - Escherichia coli

2Q80 ENSP00000351852 Geranyltranstransferase - Escherichia coli

2GDA ENSP00000343205 Glucocorticoid receptor

1V4S ENSP00000223366 Glucokinase Isoform 2 - Oryctolagus cuniculus

(Rabbit)

1NE7 ENSP00000311876 Glucosamine-6-Phosphate Isomerase -

2O28 ENSP00000216410 Glucosamine-Phosphate N-Acetyltransferase - Homo

sapiens (Human)

2BHL ENSP00000342362 Glucose-6-Phosphate 1-Dehydrogenase - Bacillus

subtilis

1YDE ENSP00000263278 Glucose 1-Dehydrogenase

2NT1 ENSP00000314508 Glucosylceramidase - Listeria monocytogenes

1KWS ENSP00000367217 Glucuronyltransferase I - Mus musculus (Mouse)

2A5T ENSP00000332549 Glutamate [NMDA] receptor subunit epsilon 2 precursor

2OKJ ENSP00000350928 Glutamate decarboxylase 1

2OKK ENSP00000365437 Glutamate decarboxylase 2

1L1F ENSP00000277865 Glutamate dehydrogenase 1, mitochondrial precursor

1YAE ENSP00000358134 Glutamate receptor, ionotropic kainate 1 precursor

1BI8 ENSP00000337056 Glutaminase, kidney isoform

2QC8 ENSP00000356537 Glutamine synthetase

1SIR ENSP00000222214 Glutaryl-Coa Dehydrogenase - Escherichia coli

2CVD ENSP00000295256 Glutathione-Requiring Prostaglandin D Syntha -

1EEM ENSP00000358727 Glutathione-S-Transferase - Homo sapiens (Human)

1YZX ENSP00000351181 Glutathione S-Transferase, Mitochondrial - Homo

sapiens (Human)

3LJR ENSP00000290765 Glutathione S-Transferase - Homo sapiens (Human)

1TDI ENSP00000211122 Glutathione S-Transferase A3-3 - Homo sapiens (Human)

3GTU ENSP00000256594 Glutathione S-Transferase Mu 1 - Homo sapiens (Human)

2HGS ENSP00000216951 Glutathione Synthetase - Mus musculus (Mouse)

1FW1 ENSP00000216465 Glutathione Transferase Zeta - Bacillus subtilis

4GR1 ENSP00000221130 Glutathione reductase (mitochondrial)

1ZNQ ENSP00000229239 Glyceraldehyde-3-phosphate dehydrogenase, liver

2PLA ENSP00000282541 Glycerol-3-phosphate dehydrogenase [NAD+],

cytoplasmic

1R74 ENSP00000361894 Glycine N-Methyltransferase - Rattus norvegicus (Rat)

2FTS ENSP00000264428 Glycine receptor beta chain

1R0E ENSP00000264235 Glycogen Synthase Kinase-3 Beta - Mus musculus

(Mouse)

2NZL ENSP00000368066 Glycolate Oxidase - Spinacia oleracea (Spinach)

2EVS ENSP00000315263 Glycolipid Transfer Protein - Trypanosoma cruzi

2HXM ENSP00000337398 Glycosylase - Simplexvirus Human herpesvirus 1 (HHV-

1)

2Q50 ENSP00000313432 Glyoxylate reductase/hydroxypyruvate reductase

2BWG ENSP00000259727 Gmp Reductase I - Saccharomyces cerevisiae (Baker's

yeast)

3HLA ENSP00000340858 Gonadotropin-releasing hormone II receptor

1PVH ENSP00000370694 Granulocyte colony stimulating factor receptor (CD114

antigen)

1IAU ENSP00000216341 Granzyme B - Trichoderma reesei (Hypocrea jecorina)

1LE7 ENSP00000261659 Group X secretory phospholipase A2

2F8Y ENSP00000277541 Growth-arrest-specific protein 6

2BHK ENSP00000363492 Growth Differentiation Factor 5 - Homo sapiens

(Human)

2HUY ENSP00000339007 Growth Factor Receptor-Bound Protein 2 - Rattus

norvegicus (Rat)

1SRQ ENSP00000363890 Gtpase-Activating Protein 1 - Methanococcus

jannaschii

1ZX0 ENSP00000252288 Guanidinoacetate N-methyltransferase

2GVZ ENSP00000360126 Guanine Nucleotide-Binding Protein G - Escherichia

coli

2Q6C ENSP00000369876 HMG-CoA Reductase

2CUD ENSP00000337548 Haematopoetic Cell Kinase - Homo sapiens (Human)

1XMM ENSP00000263579 Heat Shock-Like Protein 1 - Homo sapiens (Human)

1HJO ENSP00000364802 Heat Shock 70 Kda Protein 1 - Mus musculus (Mouse)

2UWD ENSP00000335153 Heat Shock Protein Hsp 90-Alpha - Sus scrofa (Pig)

1UYM ENSP00000329390 Heat Shock Protein Hsp 90-Beta - Sus scrofa (Pig)

1TWR ENSP00000216117 Heme oxygenase 1

2Q32 ENSP00000219700 Heme oxygenase 2

1A9W ENSP00000369586 Hemoglobin Gamma Chains -

1ZRH ENSP00000002596 Heparan Sulfate - Mus musculus (Mouse)

1T8U ENSP00000284110 Heparan Sulfate D-Glucosaminyl 3-O- Sulfotra - Mus

musculus (Mouse)

1NST ENSP00000374504 Heparan Sulfate N-Deacetylase/N-Sulfotransfe - Mus

musculus (Mouse)

2ERM ENSP00000367286 Heparin-binding growth factor 1 precursor

1JMO ENSP00000215727 Heparin cofactor II precursor

2DWY ENSP00000341344 Hepatocyte Growth Factor-Regulated Tyrosine –

Pyrococcus furiosus

1YC0 ENSP00000372224 Hepatocyte Growth Factor Activator Precursor

2G15 ENSP00000317272 Hepatocyte Growth Factor Receptor - Homo sapiens

(Human)

2LFB ENSP00000257555 Hepatocyte Nuclear Factor 1-Alpha - Synthetic

construct

1PZL ENSP00000362011 Hepatocyte Nuclear Factor 4-Alpha - Escherichia coli

1LV2 ENSP00000346339 Hepatocyte Nuclear Factor 4-Gamma - Mus musculus

(Mouse)

1ZKL ENSP00000368730 High-affinity cAMP-specific 3',5'-cyclic

phosphodiesterase 7A

2AOX ENSP00000280097 Histamine N-Methyltransferase - Rhodnius prolixus

(Triatomid bug)

1X59 ENSP00000304668 Histidyl-tRNA synthetase

2A8W ENSP00000313515 Histo-blood group ABO system transferase

2PQP ENSP00000080059 Histone Deacetylase 8 - Homo sapiens (Human)

1NW3 ENSP00000221482 Histone Methyltransferase Dot1L - Shigella sonnei

1J8F ENSP00000249396 Hst2 Protein - Archaeoglobus fulgidus

3HHR ENSP00000230882 Human Growth Hormone Receptor

1NGL ENSP00000362108 Human Neutrophil Gelatinase

1FH0 ENSP00000259470 Human Procathepsin L - T4-like viruses Bacteriophage

T4

1IVY ENSP00000361562 Human Protective Protein

1PL8 ENSP00000267814 Human Sorbitol Dehydrogenase

2HT9 ENSP00000356409 Human Thioltransferase

2PN8 ENSP00000368646 Human Thioredoxin Peroxidase-B - Gerbera hybrida

2IVV ENSP00000347942 Human keratinocyte growth factor receptor

2PE4 ENSP00000266031 Hyaluronoglucosaminidase - Streptococcus pneumoniae

1QH5 ENSP00000262303 Hydroxyacylglutathione Hydrolase - Shigella sonnei

2CW6 ENSP00000363614 Hydroxymethylglutaryl-CoA lyase

1SAW ENSP00000314622 Hypothetical 28.8 Kda Protein In Psd1-Sk -

1QZU ENSP00000343190 Hypothetical Protein Mds018 -

2J4E ENSP00000369456 Hypothetical Protein pH1917 - Pyrococcus horikoshii

1JCN ENSP00000373881 IMP dehydrogenase

1WWB ENSP00000365387 Igf-1 Receptor Kinase - Escherichia coli

2ARW ENSP00000357470 Il-6 Receptor Alpha Chain - Homo sapiens (Human)

1UCT ENSP00000347714 Immunoglobulin Alpha Fc Receptor

1XBH ENSP00000357097 Immunoglobulin E

2H32 ENSP00000375000 Immunoglobulin Heavy Chain Epsilon-1 -

2RCS ENSP00000374777 Immunoglobulin Lambda Light Chain - Homo sapiens

(Human)

2H3N ENSP00000304590 Immunoglobulin Lambda Light Chain Dimer - Homo

sapiens (Human)

1NFB ENSP00000321584 Inosine-5'-monophosphate dehydrogenase 2

2A98 ENSP00000263370 Inositol-Trisphosphate 3-Kinase A - Homo sapiens

(Human)

2QB5 ENSP00000267615 Inositol 1,3,

1N4K ENSP00000349597 Inositol 1,4,

2HHM ENSP00000256108 Inositol Monophosphatase - Homo sapiens (Human)

3GF1 ENSP00000337612 Insulin-Like Growth Factor I -

9INS ENSP00000370731 Insulin - Physeter catodon (Sperm whale)

2IFG ENSP00000357179 Insulin receptor

1JBI ENSP00000216361 Integrin Alpha-L - Homo sapiens (Human)

1MQ8 ENSP00000264832 Intercellular Adhesion Molecule-1

1ZXQ ENSP00000225760 Intercellular Adhesion Molecule-2

1AU1 ENSP00000369581 Interferon-Beta - Homo sapiens (Human)

1WLJ ENSP00000306565 Interferon Stimulated Gene 20Kda - Shigella sonnei

1FYH ENSP00000356713 Interferon gamma receptor including IFNGR1 and IFNGR2

2HYM ENSP00000371699 Interferon receptor IFNAR2c

1UC6 ENSP00000368265 Interleukin-11 receptor alpha chain (IL-11R-alpha)

1F45 ENSP00000231228 Interleukin-12 Beta Chain - Trichoderma reesei

(Hypocrea jecorina)

2ILK ENSP00000356066 Interleukin-19

1T3G ENSP00000368278 Interleukin-1 type I receptor (IL-1RI)

2B5I ENSP00000226730 Interleukin-2 - Hylobates lar (Common gibbon)

1Z92 ENSP00000369293 Interleukin-2 receptor alpha chain (IL-2-RA)

2ERJ ENSP00000216223 Interleukin-2 receptor beta chain (IL-2-RB)

1JPY ENSP00000337432 Interleukin 17F

1QX7 ENSP00000374097 Intermediate conductance Ca(2+)-activated K(+)

channel, hIK1

2GIX ENSP00000243457 Inward rectifier potassium channel 2

2CLP ENSP00000355377 Iols Protein - Mus musculus (Mouse)

1T0L ENSP00000260985 Isocitrate Dehydrogenase [Nadp] Cytoplasmic –

Escherichia coli

2ICK ENSP00000370748 Isopentenyl-Diphosphate Delta-Isomerase - Escherichia

coli

2PNY ENSP00000277517 Isopentenyl Diphosphate Delta-Isomerase - Escherichia

coli

1IVH ENSP00000249760 Isovaleryl-CoA dehydrogenase

2LVE ENSP00000374778 Kappa-4 Immunoglobulin - Homo sapiens (Human)

1IM9 ENSP00000353368 Killer Cell Immunoglobulin-Like Receptor 2Ds - Mus

musculus (Mouse)

2G1L ENSP00000346221 Kinesin-Like Protein Kif1A - Escherichia coli

2HEH ENSP00000361298 Kinesin-Like Protein Kif2C - Mus musculus (Mouse)

2UYM ENSP00000260731 Kinesin-Related Motor Protein Eg5 - Mus musculus

(Mouse)

2H58 ENSP00000368976 Kinesin Heavy Chain-Like Protein - Solanum tuberosum

2NR8 ENSP00000292334 Kinesin Heavy Chain - Mus musculus (Mouse)

2HZP ENSP00000264170 Kynureninase

1W7N ENSP00000302227 Kynurenine--oxoglutarate transaminase I

1NNL ENSP00000275605 L-3-Phosphoserine Phosphatase - Pongo pygmaeus

(Orangutan)

9JDW ENSP00000303263 L-Arginine\: Glycine Amidinotransferase - Rattus

norvegicus (Rat)

1I0Z ENSP00000229319 L-Lactate Dehydrogenase H Chain

2I6T ENSP00000323353 L-lactate dehydrogenase A-like 6A

1QIP ENSP00000362463 Lactoylglutathione Lyase - Homo sapiens (Human)

1W6J ENSP00000348762 Lanosterol Synthase - Thermus thermophilus

2P0K ENSP00000337352 Lethal - Homo sapiens (Human)

1HS6 ENSP00000228740 Leukotriene A-4 Hydrolase - Shigella sonnei

2C0C ENSP00000323678 Leukotriene B4 12- Hydroxydehydrogenase/Pros - Cavia

porcellus

1HLG ENSP00000238983 Lipase, Gastric

2GO0 ENSP00000304311 Lithostathine

2HRR ENSP00000353720 Liver Carboxylesterase I - Homo sapiens (Human)

2EX4 ENSP00000361564 Lmaj004091Aaa - Leishmania major

1RKB ENSP00000261565 Lmaj004144Aaa Protein - Leishmania major

2DM4 ENSP00000260197 Low density lipoproteins (LDL)

2O7N ENSP00000321989 Lymphocyte function-associated antigen 1 (CD11a

antigen)

1HML ENSP00000301046 Lysozyme

2NWD ENSP00000261267 Lysozyme - Pan troglodytes (Chimpanzee)

1YS0 ENSP00000245959 M-Phase Inducer Phosphatase 2 - Rattus norvegicus

(Rat)

1BY2 ENSP00000262776 Mac-2 Binding Protein

1DPT ENSP00000215773 Macrophage Migration Inhibitory Factor - Homo sapiens

(Human)

2DFD ENSP00000327070 Malate dehydrogenase, mitochondrial precursor

2C2N ENSP00000290429 Malonyl Coa: Acyl Carrier Protein Malonyltra -

1HUP ENSP00000363079 Mannose-Binding Protein A - Trichoderma reesei

(Hypocrea jecorina)

2PF5 ENSP00000243347 Mannose-Binding Protein Associated Serin - Rattus

norvegicus

1TN3 ENSP00000296130 Mannose-Binding Protein C - Trichoderma reesei

(Hypocrea jecorina)

2P3G ENSP00000356070 Map Kinase-Activated Protein Kinase 2 - Mus musculus

(Mouse)

1XU8 ENSP00000373907 Maspin Precursor - Escherichia coli

2IUH ENSP00000370749 Mast/stem cell growth factor receptor precursor

1RM8 ENSP00000286614 Matrix Metalloproteinase-16 - Homo sapiens (Human)

3USN ENSP00000299855 Matrix Metalloproteinase 3 - Homo sapiens (Human)

2DDY ENSP00000260227 Matrix metalloprotease 2 (MMP-2)

1YXK ENSP00000309124 Mdc-Sign1B Type I Isoform - Homo sapiens (Human)

2IT6 ENSP00000315477 Mdc-Sign1B Type I Isoform - Trichoderma reesei

(Hypocrea jecorina)

2A1T ENSP00000359878 Medium Chain Acyl-Coa Dehydrogenase - Escherichia

coli

2C11 ENSP00000312326 Membrane Copper Amine Oxidase

2E4Z ENSP00000373987 Metabotropic glutamate receptor 1

2NQ7 ENSP00000296411 Methionine Aminopeptidase - Escherichia coli

1B6A ENSP00000325312 Methionine Aminopeptidase - Homo sapiens (Human)

2O2K ENSP00000355536 Methionine synthase

2DJV ENSP00000262027 Methionyl-tRNA synthetase

1YFH ENSP00000302111 Methylated-DNA--protein-cysteine methyltransferase

1HYR ENSP00000331186 Mhc Class I Homolog Mic-A

2ABI ENSP00000347441 Mineralocorticoid Receptor

2P33 ENSP00000352157 Mitogen-Activated Protein Kinase 10 - Homo sapiens

(Human)

2Q3F ENSP00000358423 Molecule: Human ADP-Ribosylation Factor 1; S -

2DL4 ENSP00000273183 Molecule: Protein Kinase C Delta Type; Domain -

2BXS ENSP00000340684 Monoamine oxidase A (MAO-A)

2BDN ENSP00000225831 Monocyte Chemotactic Protein 2 - Homo sapiens (Human)

2IMG ENSP00000357089 Mrna Capping Enzyme - Shewanella oneidensis

2I99 ENSP00000219599 Mu-crystallin homolog

1GSM ENSP00000304247 Mucosal Addressin Cell Adhesion Molecule-1

2PO6 ENSP00000357153 Multiple T-cell antigens (CD1, CD2, CD3, CD5, CD5,

CD7, CD8)

2CSA ENSP00000355527 Muscarinic acetylcholine receptor M3

2DLP ENSP00000293201 Myosin II Heavy Chain Fused To Alpha-Actini -

1FSU ENSP00000342327 N-Acetylgalactosamine-4-Sulfatase

1U31 ENSP00000370939 NAD(P) transhydrogenase, mitochondrial precursor

1UMK ENSP00000338461 NADH-cytochrome b5 reductase

1S3A ENSP00000252102 NADH-ubiquinone oxidoreductase B8 subunit

2JAJ ENSP00000284031 NG,NG-dimethylarginine dimethylaminohydrolase 1

2NR1 ENSP00000360608 NMDA receptor

1QR6 ENSP00000321070 Nad-Dependent Malic Enzyme, Mitochondria - Shewanella

frigidimarina

2F1O ENSP00000319788 Nad - Homo sapiens (Human)

1B1C ENSP00000265302 Nadph-Cytochrome P450 Reductase - Escherichia coli

2QKQ ENSP00000350896 Neural Kinase, Nuk=Eph/Elk/Eck Family Recept –

Escherichia coli

2GGZ ENSP00000261047 Neuronal Calcium Sensor 1 - Thermotoga maritima

1RON ENSP00000242152 Neuropeptide Y - Rattus norvegicus (Rat)

1KR2 ENSP00000366410 Nicotinamide Mononucleotide Adenylyl Transfe –

Bacillus subtilis

2A14 ENSP00000013222 Nicotinate N-methyltransferase

1DXZ ENSP00000261008 Nicotinic acetylcholine Receptor alpha2/alpha3

2NSI ENSP00000327251 Nitric-oxide synthase, endothelial

1WMH ENSP00000219255 Nitric-oxide synthase brain

1P6F ENSP00000291890 Nk Receptor - Thermotoga maritima

2EXX ENSP00000283429 Nmra

1S0X ENSP00000261523 Nuclear Receptor Ror-Alpha - Mus musculus (Mouse)

1YUC ENSP00000254227 Nuclear receptor 0B1

2HVD ENSP00000337060 Nucleoside Diphosphate Kinase A - Mus musculus

(Mouse)

1ZS6 ENSP00000219302 Nucleoside Diphosphate Transferase - Bos taurus

(Bovine)

1R02 ENSP00000293330 Orexin-A - Homo sapiens (Human)

2OAT ENSP00000357838 Ornithine aminotransferase, mitochondrial

1OTH ENSP00000039007 Ornithine carbamoyltransferase

2OO0 ENSP00000371167 Ornithine decarboxylase

2V30 ENSP00000232607 Orotidine 5'-Phosphate Decarboxylase -

1SKX ENSP00000336528 Orphan Nuclear Receptor Pxr - Homo sapiens (Human)

1UPW ENSP00000253727 Oxysterols Receptor Lxr-Beta - Homo sapiens (Human)

1HES ENSP00000356766 P-Selectin - Homo sapiens (Human)

1Z4R ENSP00000225916 P300/Cbp Associating Factor - Escherichia coli

1BOR ENSP00000268058 PML-RAR Alpha Protein

3GWX ENSP00000353916 PPAR-alpha

1PJA ENSP00000372789 Palmitoyl-Protein Thioesterase 2 Precursor

1B2Y ENSP00000306915 Pancreatic alpha-amylase precursor

1N8S ENSP00000358223 Pancreatic triacylglycerol lipase [Precursor]

1RK9 ENSP00000216200 Parvalbumin Alpha -

2DKP ENSP00000347560 Pepp1 - Homo sapiens (Human)

1RXT ENSP00000258960 Peptide N-Myristoyltransferase - Candida albicans

(Yeast)

1JJ7 ENSP00000372596 Peptide Transporter Tap1 - Mus musculus (Mouse)

1YCK ENSP00000008938 Peptidoglycan Recognition Protein I-Alph -

2HE9 ENSP00000232978 Peptidyl-Prolyl Cis-Trans Isomerase - Homo sapiens

(Human)

2ESL ENSP00000303057 Peptidyl-Prolyl Cis-Trans Isomerase 5 - Bacillus

subtilis

2HQ6 ENSP00000370460 Peptidyl-Prolyl Cis-Trans Isomerase A - Papio anubis

(Olive baboon)

2EV8 ENSP00000358547 Peripheral Plasma Membrane Cask - Escherichia coli

1XK8 ENSP00000363609 Periplasmic Divalent Cation Tolerance Protei –

Escherichia coli

1PRX ENSP00000342026 Peroxiredoxin - Homo sapiens (Human)

1OC3 ENSP00000265462 Peroxiredoxin 5, mitochondrial precursor

1YXM ENSP00000373632 Peroxisomal Trans 2-Enoyl Coa Reductase - Homo

sapiens (Human)

2C0L ENSP00000360569 Peroxisomal multifunctional enzyme type 2

6PAH ENSP00000303500 Phenylalanine-4-hydroxylase

2G8N ENSP00000269582 Phenylethanolamine N-Methyltransferase - Homo sapiens

(Human)

2GLQ ENSP00000295450 Phosphatase

1LN3 ENSP00000268896 Phosphatidylcholine Transfer Protein - Homo sapiens

(Human)

1BEH ENSP00000261313 Phosphatidylethanolamine Binding Protein - Shewanella

oneidensis

2V1Y ENSP00000263967 Phosphatidylinositol 3-Kinase Catalytic Subu - Homo

sapiens (Human)

1W80 ENSP00000322234 Phosphatidylinositol Phosphate Phosphatase -

1UW5 ENSP00000316809 Phosphatidylinositol Transfer Protein Alpha -

2GMV ENSP00000319814 Phosphoenolpyruvate Carboxykinase, Cytosolic –

Shigella sonnei

1NUH ENSP00000348877 Phosphoglucose Isomerase - Thermococcus litoralis

2HHJ ENSP00000342032 Phosphoglycerate mutase 1

2HSP ENSP00000362369 Phosphoinositide-Specific Phospholipase C, I –

Shigella sonnei

1POE ENSP00000364252 Phospholipase A2 - Homo sapiens (Human)

2JI4 ENSP00000268835 Phosphoribosyl Pyrophosphate Synthetase - Bacillus

subtilis

2H31 ENSP00000264221 Phosphoribosylamidoimidazole- Succinocar -

1ZLY ENSP00000371261 Phosphoribosylglycinamide Formyltransferase - Homo

sapiens (Human)

1CM8 ENSP00000215659 Phosphorylated Map Kinase P38-Gamma - Escherichia

coli

2OAY ENSP00000278407 Pigment Epithelium-Derived Factor

2JOD ENSP00000269200 Pituitary Adenylate Cyclase Activating Po - Synthetic

construct

1RV6 ENSP00000238607 Placenta Growth Factor - Methanococcus jannaschii

1RBP ENSP00000360518 Plasma retinol-binding protein

2FD6 ENSP00000339328 Plasminogen

2HGF ENSP00000222390 Plasminogen precursor

1VYH ENSP00000006951 Platelet-Activating Factor Acetylhydrolas - Shigella

sonnei

2OF5 ENSP00000322314 Platelet Glycoprotein Ib Alpha Chain Precurs -

1H7U ENSP00000265849 Pms1 Protein Homolog 2

2CS1 ENSP00000343888 Pms1 Protein Homolog 2 - Mus musculus (Mouse)

1Q79 ENSP00000216277 Poly - Bos taurus (Bovine)

2PA9 ENSP00000045065 Poly [ADP-Ribose] Polymerase-1 - Homo sapiens (Human)

2D7R ENSP00000297107 Polypeptide N-Acetylgalactosaminyltransferas -

1WVO ENSP00000210444 Polysialic Acid Capsule Biosynthesis Protein -

1PE3 ENSP00000291525 Porcine Pancreatic Spasmolytic Polypepti - Homo

sapiens (Human)

2JIN ENSP00000256366 Postsynaptic Density Protein 95

2Q0A ENSP00000251287 Potassium/Sodium Hyperpolarization-Activated - Mus

musculus (Mouse)

2NZ0 ENSP00000319591 Potassium voltage-gated channel subfamily A member 1

1UJL ENSP00000346739 Potassium voltage-gated channel subfamily H member 2

2OVC ENSP00000262916 Potassium voltage-gated channel subfamily KQT member

1

2IV5 ENSP00000368752 Prion Protein

2G2J ENSP00000263576 Probable ATP-Dependent RNA Helicase P47 -

1XTJ ENSP00000372805 Probable ATP-Dependent RNA Helicase P47 - Homo

sapiens (Human)

2PL3 ENSP00000314348 Probable ATP-Dependent RNA Helicase P47 - Rattus

norvegicus (Rat)

1ZLI ENSP00000282957 Procarboxypeptidase B - Pyrococcus furiosus

1PFL ENSP00000370747 Profilin II - Pongo pygmaeus (Orangutan)

2OVM ENSP00000325120 Progesterone Receptor (PR)

1M6I ENSP00000287295 Programmed Cell Death Protein 8 - Escherichia coli

1BP3 ENSP00000231423 Prolactin receptor precursor

1TJC ENSP00000307318 Prolyl hydroxylase

2CQY ENSP00000308554 Propionyl-CoA carboxylase alpha chain

2IAG ENSP00000244043 Prostacyclin synthase

2PVW ENSP00000256999 Prostate-specific membrane antigen (7E11-C5.3

antigen/FOLH1)

2HPA ENSP00000323036 Prostatic Acid Phosphatase - Homo sapiens (Human)

2FCG ENSP00000296435 Protegrin 3 - Sus scrofa (Pig)

1KV3 ENSP00000355330 Protein-Glutamine Gamma-Glutamyltransferase -

1KR5 ENSP00000356350 Protein-L-Isoaspartate O-Methyltransferase -

2OC3 ENSP00000175756 Protein-Tyrosine-Phosphatase - Homo sapiens (Human)

2QEP ENSP00000374070 Protein-Tyrosine Phosphatase, Non-Receptor T - Homo

sapiens (Human)

2NZ6 ENSP00000278456 Protein-Tyrosine Phosphatase 1B - Homo sapiens

(Human)

2FYT ENSP00000329586 Protein Arginine Methyltransferase Prmt3 -

1ORI ENSP00000246789 Protein Arginine N-Methyltransferase 1 -

2UZP ENSP00000263431 Protein Kinase C, Theta Type - Methanococcus

jannaschii

2ONI ENSP00000350569 Protein Kinase C - Rattus norvegicus (Rat)

1KPF ENSP00000304229 Protein Kinase C Interacting Protein - Homo sapiens

(Human)

2P5X ENSP00000370718 Protein Maf - Bacillus subtilis

1LL8 ENSP00000234040 Proto-Oncogene Serine/Threonine-Protein - Escherichia coli

2OBJ ENSP00000362608 Proto-Oncogene Serine/Threonine-Protein Kina - Homo sapiens (Human)

2SRC ENSP00000362683 Proto-Oncogene Tyrosine-Protein Kinase S - Homo sapiens (Human)

2C9W ENSP00000339428 Proto-Oncogene Tyrosine-Protein Kinase Src -

2HZI ENSP00000361423 Proto-oncogene tyrosine-protein kinase ABL1

1M8Z ENSP00000257075 Pumilio 1 - Rattus norvegicus (Rat)

2ON6 ENSP00000354532 Purine nucleoside phosphorylase

2BQ8 ENSP00000218758 Purple Acid Phosphatase

1TG6 ENSP00000245816 Putative ATP-Dependent Clp Protease Proteoly –

Thermotoga maritima

2F8N ENSP00000353806 Putative Polyprotein/Phosphatase - Homo sapiens

(Human)

1D3B ENSP00000342305 Putative Snrnp Sm-Like Protein -

2HW1 ENSP00000369642 Putative Sugar Kinase - Salmonella typhimurium lt2

2F7K ENSP00000291565 Pyridoxal kinase

2CFT ENSP00000215904 Pyridoxal phosphate phosphatase

1NRG ENSP00000225573 Pyridoxine-5'-phosphate oxidase

2IZZ ENSP00000328858 Pyrroline-5-carboxylate reductase 1

2H5G ENSP00000360268 Pyrroline-5-carboxylate synthetase

2Q8I ENSP00000368460 Pyruvate Dehydrogenase Kinase, Isozyme 2 - Mus

musculus (Mouse)

2OZL ENSP00000307241 Pyruvate dehydrogenase

2BEW ENSP00000269980 Pyruvate dehydrogenase E1 component alpha subunit,

2COO ENSP00000359150 Pyruvate dehydrogenase protein X component,

mitochondrial precursor

1ZJH ENSP00000373744 Pyruvate kinase, isozymes M1/M2

1LIU ENSP00000339933 Pyruvate kinase, isozymes R/L

2NT2 ENSP00000269033 Pyst1 - Methanococcus jannaschii

2OBY ENSP00000337834 Quinone Oxidoreductase - Shigella sonnei

2QR2 ENSP00000369822 Quinone Reductase Type 2 - Escherichia coli

2UVM ENSP00000270202 RAC serine/threonine-protein kinase

1RFA ENSP00000251849 RAF proto-oncogene serine/threonine-protein kinase

2OR3 ENSP00000340278 RNA-Binding Protein Regulatory Subunit –

Staphylococcus aureus

2YVC ENSP00000342830 Radixin - Rattus norvegicus (Rat)

1WA5 ENSP00000254675 Ran - Triticum aestivum (Wheat)

1RYH ENSP00000348461 Ras-Related C3 Botulinum Toxin Substrate 1 I - Homo

sapiens

2OIL ENSP00000338772 Ras-Related Protein Rab-11A - Triticum aestivum

(Wheat)

2IWR ENSP00000328160 Ras-Related Protein Rab-9A - Triticum aestivum

(Wheat)

2NZJ ENSP00000201979 Ras-Related Protein Ral-A - Triticum aestivum (Wheat)

3RAB ENSP00000222256 Ras-Related Protein Sec4 - Saccharomyces cerevisiae

(Baker's yeast)

2P0A ENSP00000330219 Rat Synapsin I - Mus musculus (Mouse)

1M6B ENSP00000267101 Receptor Protein-Tyrosine Kinase Erbb-3

1ITU ENSP00000261615 Renal Dipeptidase - Homo sapiens (Human)

2V12 ENSP00000356164 Renin

1P8T ENSP00000043402 Reticulon 4 Receptor

2FF0 ENSP00000362690 Retinoic acid receptor RXR-alpha

1UHL ENSP00000372563 Retinoic acid receptor RXR-beta

2GL8 ENSP00000356867 Retinoic acid receptor RXR-gamma

1DSZ ENSP00000316769 Retinoic acid receptor alpha

1XAP ENSP00000373282 Retinoic acid receptor beta

4LBD ENSP00000343698 Retinoic acid receptor gamma-2

2BO9 ENSP00000264265 Retinoic acid receptor responder protein 1

2CLS ENSP00000308461 Rho-Related GTP-Binding Protein Rhoe - Rattus

norvegicus (Rat)

1Q9S ENSP00000257452 Riboflavin kinase

2FV7 ENSP00000368978 Ribokinase - Escherichia coli

1ONI ENSP00000254878 Ribonuclease UK114

2NN6 ENSP00000315476 Ribonuclease pH - Paracoccus pantotrophus

(Thiosphaera pantotropha)

2UW2 ENSP00000353770 Ribonucleoside-Diphosphate Reductase M2 Chai -

2C9O ENSP00000318297 Ruvb - Homo sapiens (Human)

2BCX ENSP00000354254 Ryanodine receptor 1

1LI4 ENSP00000217426 S-Adenosylhomocysteine Hydrolase - Homo sapiens

(Human)

1JEN ENSP00000357880 S-adenosylmethionine decarboxylase proenzyme

2P02 ENSP00000303147 S-adenosylmethionine synthetase

3PSR ENSP00000357712 S100A6 - Rattus norvegicus (Rat)

1OLM ENSP00000316203 SEC14-like protein 2

1NHL ENSP00000249647 SNAP-25

2GAO ENSP00000362339 Sar1 - Triticum aestivum (Wheat)

1WPE ENSP00000349595 Sarcoplasmic/Endoplasmic Reticulum Calcium A –

Escherichia coli

2BOX ENSP00000319454 Secretin receptor

2H1K ENSP00000370421 Segmentation Polarity Homeobox Protein Engrai -

1Z6Z ENSP00000234454 Sepiapterin Reductase - Rana perezi (Perez's frog)

1H0C ENSP00000302620 Serine--pyruvate aminotransferase

2NP8 ENSP00000216911 Serine/Threonine Kinase 6 - Mus musculus (Mouse)

2V3S ENSP00000311713 Serine/Threonine Protein Kinase Tao2 - Rattus

norvegicus

1WAO ENSP00000012443 Serine/Threonine Protein Phosphatase 5 –

Methanococcus jannaschii

1FJM ENSP00000365936 Serine/Threonine Protein Phosphatase Pp1-Gam -

1VJY ENSP00000364133 Serine/threonine-protein kinase receptor R2

1BJ4 ENSP00000318868 Serine hydroxymethyltransferase, cytosolic

2A7V ENSP00000333667 Serine hydroxymethyltransferase (mitochondrial)

2HAV ENSP00000264998 Serotransferrin precursor

2I30 ENSP00000295897 Serum albumin precursor

2F69 ENSP00000274031 Set9 - Saccharomyces cerevisiae (Baker's yeast)

1LHW ENSP00000369816 Sex hormone-binding globulin precursor

1U6T ENSP00000362308 Sh3 Domain-Binding Glutamic Acid-Rich Protein -

2SHP ENSP00000340944 Shp-2 - Homo sapiens (Human)

2A25 ENSP00000349156 Siah-1A Protein - Rattus norvegicus (Rat)

2F29 ENSP00000233840 Sialidase 2 - Respirovirus Human parainfluenza 3

virus (HPIV-3)

1RY1 ENSP00000305230 Signal Recognition Particle 9/14 Fusion Prot -

2J37 ENSP00000216774 Signal Recognition Particle Protein - Escherichia

coli

2GO5 ENSP00000328023 Signal Sequence Recognition Protein Ffh -

2NYR ENSP00000368552 Silent Information Regulator 2 - Archaeoglobus

fulgidus

3ULL ENSP00000265304 Single Stranded DNA Binding Protein - Escherichia

coli

1YGS ENSP00000341551 Smad2 - Methanococcus jannaschii

2HCI ENSP00000351671 Small Inducible Cytokine A20 - Methanococcus

jannaschii

1RJT ENSP00000306884 Small Inducible Cytokine B10 - Homo sapiens (Human)

2HCC ENSP00000293277 Small inducible cytokine A23 precursor

2HTG ENSP00000263980 Sodium/hydrogen exchanger 1

2H9G ENSP00000317859 Soluble Tumor Necrosis Factor Receptor 1 - Homo

sapiens (Human)

2I4K ENSP00000326668 Sorting Nexin Grd19 - Escherichia coli

2O0L ENSP00000366156 Spermidine synthase

2OHF ENSP00000284719 Spo0B-Associated GTP-Binding Protein - Bacillus

subtilis

1WBP ENSP00000339450 Sr Protein Kinase - Mus musculus (Mouse)

1YVL ENSP00000354394 Stat Protein - Dictyostelium discoideum

1P49 ENSP00000217961 Steryl-Sulfatase - Homo sapiens (Human)

2AII ENSP00000272902 Sulfatase Modifying Factor 2 - Homo sapiens (Human)

1MJ4 ENSP00000266971 Sulfite Oxidase - Pseudomonas stutzeri (Pseudomonas

perfectomarina)

2H8K ENSP00000333310 Sulfotransferase - Mus musculus (Mouse)

1Q22 ENSP00000201586 Sulfotransferase Family, Cytosolic, 2B, Memb - Mus

musculus (Mouse)

1DO5 ENSP00000307870 Superoxide Dismutase - Shigella sonnei

2V0A ENSP00000374645 Superoxide Dismutase [Cu-Zn] - Mycobacterium

tuberculosis

2ADQ ENSP00000356022 Superoxide Dismutase [Mn],

2OQ5 ENSP00000307519 Suppressor Of Tumorigenicity 14 - Homo sapiens

(Human)

1XIW ENSP00000354566 T-cell surface glycoprotein CD3 epsilon chain (CD3E)

1RSF ENSP00000303395 T Lymphocyte Activation Antigen

1W4M ENSP00000234313 Tandem pH Domain Containing Protein-1 - Pyrococcus

furiosus

2BKA ENSP00000344925 Tat-Interacting Protein Tip30 - Homo sapiens

2B69 ENSP00000283148 Tdp-Glucose-4,6-Dehydratase

2J64 ENSP00000270879 Techylectin-5A

2HTF ENSP00000242248 Terminal Deoxynucleotidyltransferase Short I - Mus

musculus (Mouse)

2H64 ENSP00000340361 Tgf-Beta Receptor Type I - Rattus norvegicus (Rat)

2H8L ENSP00000370947 Thioredoxin - Chain B - Methanococcus jannaschii

2IIY ENSP00000363641 Thioredoxin - Shigella sonnei

2J3N ENSP00000373506 Thioredoxin reductase - Homo sapiens (Human)

1WWT ENSP00000371549 Threonyl-tRNA synthetase

8KME ENSP00000374561 Thrombin

2ADX ENSP00000366307 Thrombomodulin

1YO8 ENSP00000355751 Thrombospondin 1 - Homo sapiens (Human)

2OT3 ENSP00000370208 Thromboxane A2 receptor

1XBT ENSP00000301634 Thymidine Kinase, Cytosolic - Homo sapiens (Human)

2J0F ENSP00000252029 Thymidine Phosphorylase - Homo sapiens (Human)

1NN5 ENSP00000304802 Thymidylate Kinase - Homo sapiens (Human)

2ONB ENSP00000315644 Thymidylate synthase

2H79 ENSP00000264637 Thyroid hormone receptor alpha

2H6W ENSP00000280696 Thyroid hormone receptor beta-2

1MYP ENSP00000225275 Thyroid peroxidase

2J67 ENSP00000354459 Toll-Like Receptor 2 - Homo sapiens (Human)

1SZ7 ENSP00000362261 Trafficking Protein Particle Complex Subunit - Mus

musculus (Mouse)

1F05 ENSP00000321259 Transaldolase - Shigella flexneri

2BB5 ENSP00000215838 Transcobalamin II

4PRG ENSP00000312472 Transcription factor PPAR gamma

2NSU ENSP00000353224 Transferrin Receptor Protein

2TGI ENSP00000355897 Transforming Growth Factor-Beta 3 - Rattus norvegicus

(Rat)

2PJH ENSP00000351777 Transitional Endoplasmic Reticulum Atpas - Mus

musculus (Mouse)

2DVW ENSP00000157812 Transitional Endoplasmic Reticulum Atpase - Mus

musculus (Mouse)

2ROY ENSP00000237014 Transthyretin precursor

1SMO ENSP00000244709 Triggering Receptor Expressed On Myeloid Cel –

Escherichia coli

2FRG ENSP00000362219 Triggering Receptor Expressed On Myeloid Cel - Homo

sapiens (Human)

2IAN ENSP00000371705 Triosephosphate Isomerase -

1OZS ENSP00000341838 Troponin I, cardiac muscle

1J1E ENSP00000356288 Troponin T, cardiac muscle

1MLW ENSP00000343550 Tryptophan 5-hydroxylase 1

2DR2 ENSP00000347495 Tryptophanyl-tRNA synthetase

2FTC ENSP00000225969 Tt0826 - Bacillus subtilis

2FIM ENSP00000319414 Tubby Protein - Mus musculus

1Z5W ENSP00000251413 Tubulin beta chain

2QE3 ENSP00000363157 Tumor necrosis factor alpha

1QLY ENSP00000308176 Tyrosine-Protein Kinase Btk - Homo sapiens (Human)

2EVA ENSP00000358335 Tyrosine-Protein Kinase Itk/Tsk - Homo sapiens

(Human)

1RHF ENSP00000263798 Tyrosine-Protein Kinase Receptor Tyro3 - Shigella

sonnei

2FCI ENSP00000364907 Tyrosine-Protein Kinase Syk - 1-

2OQ1 ENSP00000264972 Tyrosine-Protein Kinase Zap-70 - Homo sapiens (Human)

2EFK ENSP00000320117 Tyrosine-protein kinase ABL2

2HNQ ENSP00000360683 Tyrosine-protein phosphatase, non-receptor type 1

5PNT ENSP00000371762 Tyrosine Phosphatase - Aeropyrum pernix

1Q11 ENSP00000362576 Tyrosyl-tRNA synthetase, cytoplasmic

2FY1 ENSP00000372104 U1A RNA Binding Domain - Shigella flexneri

2QG4 ENSP00000371259 UDP-glucose 6-dehydrogenase

1TEV ENSP00000360939 UMP-CMP kinase

2JG4 ENSP00000265986 Ubiquinol-cytochrome-c reductase complex core protein

I,

2O25 ENSP00000373626 Ubiquitin-Conjugating Enzyme E2-25 Kda - Rattus

norvegicus (Rat)

2OB4 ENSP00000215574 Ubiquitin-Conjugating Enzyme E2 2 - Caenorhabditis

elegans

2NVU ENSP00000250495 Ubiquitin-Like Protein 7, Rub1 - Thermotoga maritima

2HDP ENSP00000258149 Ubiquitin-Protein Ligase E3 Mdm2 - Homo sapiens

(Human)

2PEA ENSP00000272317 Ubiquitin - Xenopus laevis (African clawed frog)

1EK6 ENSP00000313026 Udp-Galactose 4-Epimerase

1JVG ENSP00000271469 Udpglcnac Pyrophosphorylase - Escherichia coli

2UVQ ENSP00000361289 Uridine-Cytidine Kinase 2 - Mus musculus (Mouse)

1VJA ENSP00000361847 Urokinase-Type Plasminogen Activator - Homo sapiens

(Human)

1URO ENSP00000246337 Uroporphyrinogen Decarboxylase - Homo sapiens (Human)

1PBU ENSP00000367258 Valyl-tRNA synthetase

2VPF ENSP00000361099 Vascular endothelial growth factor

1Y6B ENSP00000370742 Vascular endothelial growth factor receptor 2

precursor

1YTV ENSP00000299178 Vasopressin V1a Receptor

1IVT ENSP00000357277 Vimentin - Shigella sonnei

1MA9 ENSP00000273951 Vitamin D Binding Protein -

1KB6 ENSP00000229022 Vitamin D Receptor

2F3Y ENSP00000266376 Voltage-dependent L-type calcium channel alpha-1C

subunit

2D46 ENSP00000343563 Voltage-dependent L-type calcium channel beta-1

subunit

2OEW ENSP00000307387 Voltage-dependent P/Q-type calcium channel alpha-1A

subunit

1BYY ENSP00000353206 Voltage gated sodium channel

2FF3 ENSP00000223023 Wiskott-Aldrich Syndrome Protein - Homo sapiens

2OKN ENSP00000244137 Xaa-Pro Aminopeptidase - Escherichia coli

2CKJ ENSP00000368727 Xanthine oxidase

1ZAG ENSP00000292401 Zinc-Alpha-2-Glycoprotein

2ESV ENSP00000372943 Zinc-Alpha-2-Glycoprotein - Trichoderma reesei

(Hypocrea jecorina)

2I6B ENSP00000361819 adenosine kinase

2FM5 ENSP00000345502 cAMP Phosphodiesterase

2H44 ENSP00000347046 cGMP-specific 3',5'-cyclic phosphodiesterase

2OUY ENSP00000355847 cGMP Phosphodiesterase

2JCM ENSP00000358887 cytosolic 5- nucleotidase II

2JBH ENSP00000318602 hypoxanthine-guanine phosphoribosyltransferase

1L6J ENSP00000361405 matrix metalloprotease 9 (MMP-9)

2BRQ ENSP00000267082 platelet receptor glycoprotein IIIa

1U8C ENSP00000261023 platelet receptor glycoprotein IIb

1OU5 ENSP00000280591 tRNA Cca-Adding Enzyme - Bacillus stearothermophilus
