# Supplementary material for: In Silico Elucidation of the Molecular Mechanism Defining the Adverse Effect of Selective Estrogen Receptor Modulators
Source: PLoS Comput Biol. 2007 Nov 30;3(11):e217. doi: 10.1371/journal.pcbi.0030217 (PMC2098847; doi:10.1371/journal.pcbi.0030217)
Supplement: Table S2 — Top-ranked proteins that belong to the nuclear receptor fold are not listed because they share the same fold as the template ERα (1.0 MB TIF) [file pcbi.0030217.st002.doc]

**Table S2. Top ten off-fold hits other than SERCA found by searching the ERα ligand binding site using SOIPPA. Top ranked proteins that belong to the nuclear receptor fold are not listed because they share the same fold as the template ERα.**

| **PDB Id** | **Protein** | **p-value** |
| --- | --- | --- |
| 2OCC | Heart Cytochrome C oxidase | 1.1e-3 |
| 2HR7 | Insulin receptor | 2.2e-2 |
| 1TT1 | Glutamate receptor, ionotropic kainate 2 | 2.7e-2 |
| 1ZOP | Integin CD11A | 2.7e-2 |
| 1O9K | Retinoblastoma tumor suppressor protein | 3.2e-2 |
| 2HK5 | Rubrerythrin | 3.8e-2 |
| 1Z5W | Tubulin gamma-1 | 6.2e-2 |
| 2P3G | MAP kinase-activated protein kinase 2 | 7.8e-2 |
| 1TAZ | Nucleotide phosphodiesterase B | 1.0e-1 |
| 1RXT | Glycylpeptide N-tetradecanoyl transferase | 1.1e-1 |
